# Influence of bradykinin B2 receptor and dopamine D2 receptor on the oxidative stress, inflammatory response, and apoptotic process in human endothelial cells

**DOI:** 10.1371/journal.pone.0206443

**Published:** 2018-11-14

**Authors:** Anna Niewiarowska-Sendo, Andrzej Kozik, Ibeth Guevara-Lora

**Affiliations:** Department of Analytical Biochemistry, Faculty of Biochemistry, Biophysics and Biotechnology, Jagiellonian University in Krakow, Kraków, Poland; University of PECS Medical School, HUNGARY

## Abstract

Endothelial dysfunction is a hallmark of a wide range of cardiovascular diseases and is often linked to oxidative stress and inflammation. Our earlier study reported the formation of a functional heterodimer between bradykinin receptor 2 (B2R) and dopamine receptor 2 (D2R) that may modulate cell responses, dependent on intracellular signaling. Here, for the first time, we showed a cooperative effect of these receptors on the modulation of processes involved in oxidative stress, inflammation, and apoptosis in endothelial cells. Sumanirole, a specific D2R agonist, was shown to diminish the excessive production of reactive oxygen species induced by bradykinin, a proinflammatory B2R-activating peptide. This effect was accompanied by modified activities of antioxidant enzymes and increased phosphorylation of endothelial nitric oxide synthase, leading to enhance NO production. In turn, endothelial cell co-stimulation with B2R and D2R agonists inhibited the release of interleukin-6 and endothelin-1 and modulated the expression of apoptosis markers, such as Bcl-2, Bcl-xL, Bax, and caspase 3/7 activity. All these observations argue that the D2R agonist counteracts the pro-oxidative, pro-inflammatory, and pro-apoptotic effects induced through B2R, finally markedly improving endothelial functions.

## Introduction

Many endothelial dysfunctions are closely associated with oxidative stress generation. A large body of evidence has indicated that reactive oxygen species (ROS) participate in disorders such as hypertension, hypercholesterolemia, and atherosclerosis. Enhanced oxidative stress may impair endothelium-dependent vascular relaxation and induce vascular contractile activity [1–2]. The importance of oxidative stress in the appearance of chronic heart failure has also been documented. Rapid production of ROS after heart failure can overwhelm antioxidant defenses and cause further tissue damage [3]. Moreover, augmented ROS release can lead to pathological angiogenesis, as observed during cancer progression, by modulation of the vascular endothelial growth factor production [4]. Therefore, studies involving new antioxidant mechanisms in the regulation of endothelial dysfunction may be of interest.

Bradykinin (BK), a nonapeptide rapidly produced and degraded under physiological conditions at vessel walls, plays an essential role in numerous processes occurring in the endothelium [5]. The biological effects of bradykinin are mainly mediated by the bradykinin receptor type 2 (B2R), which belongs to the large superfamily of G protein-coupled receptors (GPCRs). B2R activation is particularly important in the regulation of vascular tone and arterial pressure [5]. However, a high concentration of this peptide can modify various endothelial functions, e.g., by increasing vascular permeability and inducing angiogenesis [6], i.e. processes that are accompanied by the release of proinflammatory mediators and strictly correlated with the development of oxidative stress [7]. The precise role of BK in the regulation of oxidative stress is still not clear. Numerous studies have suggested that this peptide acts as an antioxidative factor. Such a protective role of BK is manifested by suppression of ROS production and an increase in superoxide dismutase (SOD) activity in endothelial progenitor cells as well as in cardiomyocytes [8–9]. On the other hand, it has also been shown that BK can induce ROS generation in endothelial cells and vascular smooth muscle cells [10–12]. Furthermore, BK can increase the release of F2-isoprostane in patients, leading to a strong pro-oxidative response in the human vasculature [13].

The dopamine receptor type 2 (D2R), another member of the GPCR superfamily, is also involved in the regulation of the balance between ROS generation and antioxidant systems [14]. The fact that D2R agonists exert neuroprotective effects by activating antioxidant and anti-apoptotic processes is well known [15]. It has also been demonstrated that the D2R agonist ropinirole decreases lipid peroxidation and modulates catalase (CAT) and superoxide dismutase activities in the mice striatum [16]. In contrast, injection of the D2R antagonist can abolish the antioxidant effect of this receptor in the rat brain [17]. The dopamine D2 receptor is present in several cell types including endothelial cells, in which it regulates diverse functions. The importance of this receptor in down-regulation of von Willebrand factor secretion, resulting in a reduction of endothelial activation during inflammation, has been reported [18]. In addition, D2R stimulation increases the expression of endogenous antioxidants including the paraoxonase enzyme, which is responsible for prevention of endothelial cell apoptosis [19]. These findings suggest that D2R agonists may be useful in regulating disorders that involve endothelium dysfunction.

Lately, there has been growing interest in cooperation between GPCRs, particularly in the context of their oligomerization, which may be associated with the regulation of physiological processes through changes in signaling pathways of each receptor [20]. An appreciable number of interactions of B2R and D2R with other GPCRs to form oligomeric complexes has been recently reported (for a review see reference [21]). Furthermore, we have reported for the first time that the formation of a functional heterodimer between B2 and D2 receptors can modulate cell responses and that this is dependent on the intracellular Ca^2+^concentration as well as on cAMP-dependent pathways [22]. This recent evidence has thus led us to suppose that the interaction between B2R and D2R can have consequences for the regulation of oxidative and inflammatory processes in endothelial cells. Hence, we here investigated the influence of BK and sumanirole (SUM), i.e. specific agonists for the B2 and D2 receptors, respectively, on oxidative stress, inflammatory response, and apoptotic processes in human endothelial cells.

## Materials and methods

### Chemicals

BK was supplied by Bachem (Bubendorf, Switzerland). Antibiotics, antimycotics, cell culture medium, and fetal bovine serum (FBS) were purchased from Corning (Corning, NY). All primers were obtained from Genomed (Warsaw, Poland). Apocynin, bacitracin, captopril, 4',6-diamidyno-2-fenyloindol (DAPI), 2,3-diaminonaphthalene, 2,7-dichlorodihydrofluorescein diacetate (DCFH-DA), endothelial cell growth supplement, heparin, icatibant (HOE 140), 2-mercaptomethyl-3-guanidinoethylthiopropanoic acid, nitro blue tetrazolium, protease inhibitors, SUM maleate, S-(−)-eticlopride hydrochloride (eticlopride), and standard chemicals were supplied by Sigma-Aldrich (St Louis, MO). The human tumor necrosis factor alpha (TNF-α) was purchased from Thermo Fisher Scientific (Waltham, MA).

### Antibodies

Mouse monoclonal antibody raised against Bcl-2 (#15071) and rabbit monoclonal antibody against Bcl-xL (#2764) were obtained from Cell Signaling Technology (Danvers, MA). Rabbit polyclonal antibody against the D2 receptor (MBS612859) was purchased from MyBioSource (San Diego, CA) and rabbit polyclonal antibody against the B2 receptor (NBP2-14351) from Novus Biologicals (Littleton, CO). Mouse monoclonal antibodies against β-actin (MAB8929) and Bax (MAB846) as well as goat polyclonal antibody against mouse or rabbit IgG conjugated with horseradish peroxidase (HAF007 or HAF008, respectively) were supplied by R&D Systems (Minneapolis, MN). Mouse monoclonal antibody against endothelial nitric oxide synthase (NOS3) (sc-376751) and against phospho-NOS3 (pNOS3) (sc-293032) were purchased from Santa Cruz Biotechnology (Dallas, TX). Goat polyclonal antibody against rabbit IgG conjugated with Alexa-Fluor488 (A11008) was obtained from Thermo Fisher Scientific.

### Cell culture

The human endothelial cell line (HUV-EC-C; ATCC CRL-1730) purchased from ATCC (Manassas, VA) was cultured in F-12K medium (Kaighn's Modification of Ham's F-12 Medium) supplemented with 10% FBS, 1 U/mL penicillin, 1 μg/mL streptomycin, 2.5 μg/mL amphotericin B, 50 μg/ml endothelial cell growth supplement, and 100 μg heparin at 37°C in a humidified atmosphere containing 5% CO_2_. Twenty-four hours before each experiments, cells were cultured in cell medium with 10% FBS without growth factors and all stimulations were performed in F-12K medium containing 1% FBS.

### Gene expression analysis

Total RNA from cells was isolated with ReliaPrep RNA Miniprep Systems (Promega, Madison, WI) and cDNA was synthesized with an M-MLV Reverse Transcriptase kit (Promega) in accordance with the manufacturer’s instructions. First, cDNA was synthesized from 500 ng of total RNA. Next, 2 μl of cDNA was amplified in a 25 μl reaction mixture with PCR Master Mix 2x (Thermo Fisher Scientific) and specific primers using a C1000 Touch Thermal Cycler (Bio-Rad, Hercules, CA). The primer pair sequences for B2R were 5’-TGCTGCTGCTATTCATCATC-3’ (sense) and 5’-CCAGTCCTGCAGTTTGTGAA-3’ (antisense), which are complementary to template at positions 1020–1039 and 1335–1354, respectively. Whereas primer pair for D2R were 5’-CTCTTCGGACTCAATAACGCA-3’ (sense) and 5’-CTTTAGTGGAGCCCTCAGGT-3’ (antisense) which are complementary to template at positions 746–766 and 939–958, respectively. The B2R primers are located in the exon 3 while D2R primers are located between exons 3 and 4. The PCR conditions were as follows: 95°C for 10 min followed by 30 cycles of 95°C for 15 s, 58°C for 15 s, and 72°C for 30 s. The PCR products for B2R and D2R, resolved on a 2% agarose gel, were 335 and 213 bp in length, respectively.

### Detection of B2 and D2 receptor protein expression by immunofluorescence

The cell surface expression of B2R and D2R was examined by immunofluorescence microscopy using antibodies against external receptor epitopes. Briefly, cells (3 × 10^5^) were seeded onto glass coverslips in 12-well plates, fixed in 3.7% paraformaldehyde, and incubated in 10% FBS for one hour. The plates were then incubated overnight with primary antibodies (1:250 dilution) at 4°C. After washing, the samples were treated for one hour with the secondary antibody conjugated with Alexa-Fluor488 (1:250 dilution). Then, 2 μM DAPI was added for 10 minutes and, after extensive washing, the cover slides were mounted on microscope slides using Fluorescent Mounting Medium (DAKO, Santa Clara, CA). A negative control sample with no primary antibody incubation was also prepared. The samples were analyzed with an epifluorescence microscope (Leica DMI6000B, Wetzlar, Germany) at a magnification of 40× with oil immersion.

### ROS measurement

The intracellular ROS level was determined using a fluorometric assay with DCFH-DA dye [23]. Cells (1 × 10^5^) were seeded onto a microplate precoated with 1% gelatin. After a 24-hour starvation, the cells were incubated for 30 minutes with 100 μM DCFH-DA in medium containing 1% FBS. After washing, cell stimulation with 100 nM BK, 100 nM SUM, or with both agonists was performed. The fluorescence signal changes were measured every 30 seconds over a 20-minute period at 37°C, using a microplate Synergy H1 reader (BioTek Instruments, Winooski, VT) at excitation and emission wavelengths of 485 nm and 528 nm, respectively. Some samples were pretreated with the B2R and D2R antagonists for 30 minutes (10 μM HOE 140 or 10 μM eticlopride, respectively) or for one hour with 1 μM apocynin before dye loading. The percentage changes in ROS production were presented relative to the untreated cells, which were assigned a reference value of 100%.

### Analysis of the antioxidant enzyme activity

Changes in catalase activity were evaluated by spectrometric measurements of hydrogen peroxide decomposition [24]. Endothelial cells (1 × 10^6^) were stimulated with 100 nM BK, 100 nM SUM, or both agonists, and after 2-hour incubation CAT activity in cell lysates was analyzed using a Hitachi U-2910 spectrophotometer (Tokyo, Japan). In some experiments, cells were preincubated with 10 μM HOE 140 or eticlopride for 30 minutes before stimulation. In turn, superoxide dismutase activity was measured with an in-gel activity assay after resolution of cell lysate proteins by native PAGE. Enzymatic activity was detected by the reduction of nitro blue tetrazolium in the presence of riboflavin and light [25]. Mitochondrial (MnSOD) and cytoplasmic (Cu/ZnSOD) superoxide dismutase activities were measured by densitometric estimation of stained SOD-generated products with Quantity One Software (BioRad, CA, USA). Changes in CAT an SOD activity were normalized to the total protein level in each sample, which was determined using the Bradford method.

### Measurement of NO release

NO release was measured as described previously [26]. Briefly, cells (4 × 10^5^) were seeded into a 12-well plate. After achieving 80% confluence and starvation, the cells were treated with 100 nM BK, 100 nM SUM, or both agonists simultaneously for 30 and 60 minutes. After incubation, supernatant fluorescence was measured using a Synergy H1 microplate reader at the excitation and emission wavelengths of 365 nm and 450 nm, respectively. The NO production was normalized to the amount of sample protein determined by the Lowry method.

### Detection of intracellular proteins by western blotting

Cells (1 × 10^6^) were treated with 100 nM BK, 100 nM SUM, or both agonists for 6 and 24 hours for the determination of Bax, Bcl-2, and Bcl-xL protein levels, or for 2 and 5 minutes for the measurement of endothelial nitric oxide synthase (NOS3) and NOS3 phosphorylated at Ser1177 (pNOS3). Thereafter, cell lysates were prepared with Laemmli lysis buffer (0.125 M Tris-HCl, 20% glycerol, 4% SDS, 400 mM dithiothreitol, 0.004% bromophenol blue, pH 6.8) supplemented with 5 mM sodium fluoride and 1 mM sodium orthovanadate. Equal volumes of each sample were subjected to SDS-PAGE (polyacrylamide gel electrophoresis) in a 12% gel. Resolved proteins were transferred to polyvinylidene difluoride membranes (Sigma Aldrich) in Tris/glycine buffer (25 mM Tris, 192 mM glycine, 20% methanol, pH 8.3) for 1 hour. After overnight blocking with 5% non-fat milk, the membrane was incubated for 24 hours at 4°C with primary antibodies at dilutions of 1:200 for anti-pNOS3 and anti-NOS3, 1:500 for anti-Bcl-2, 1:1000 for anti-Bax, and 1:2000 for anti-β-actin and anti-Bcl-xL. Next, after extensive washing, the membrane was incubated with the respective secondary antibody conjugated with horseradish peroxidase at a 1:1000 dilution. After one-hour incubation and extensive washing, enzymatic reaction was performed with Immobilon Western Chemiluminescent HRP Substrate (Sigma-Aldrich) in accordance with the manufacturer’s instructions. β-actin, as a housekeeping protein, was analyzed in every blot as a normalization control. The bands was analyzed by densitometric analysis with Quantity One Software.

### Measurement of interleukin-6 concentration by ELISA

Confluent cells seeded into a 12-well plate were stimulated for 6 hours with 100 nM BK, 100 nM SUM, or both agonists. Some samples were additionally preincubated with 10 μM HOE 140 or 10 μM eticlopride for 30 minutes prior to receptor agonist induction. All experiments were performed in cell medium supplemented with protease and kininase inhibitors (10 μM 2-mercaptomethyl-3-guanidinoethylthiopropanoic acid, 20 μM captopril, and 500 μM bacitracin). The interleukin-6 (IL-6) concentration in the medium was evaluated using a specific ELISA kit (BD Biosciences, Franklin Lakes, NJ) in accordance with the manufacturer’s instructions and normalized to the amount of sample protein. Absorbance was measured with a PowerWave microplate reader (BioTek Instruments) at 450 nm. An inflammated cellular model was established using cells pretreated with the inflammatory mediator TNF-α at a concentration of 10 ng/ml for 24 hours before stimulation with agonists.

### Caspase 3/7 activity measurements

Caspase 3/7 activity was assayed with a chemiluminescent CaspoGlo3 assay (Promega) using a luminogenic substrate. Cells (1 × 10^4^) were preincubated or not with 10 ng/ml TNF-α for 6 hours. Next, the cells were stimulated with receptor agonists, as mentioned above, in 1% FBS cell medium supplemented with kininase inhibitors. After 24 hours, caspase 3/7 activity was studied according to the manufacturer’s instructions. Chemiluminescence was measured using a Synergy H1 microplate reader. The obtained values in the samples are presented as percentage changes in the chemiluminescence signal relative to a control sample (untreated cells), which was assigned a reference value of 100%.

### Analysis of endothelin-1 production by ELISA

Confluent cells seeded into a 12-well plate were pretreated or not with 10 ng/ml TNF-α for 6 hours and subsequently stimulated for 24 hours with 100 nM BK, 100 nM SUM, or both agonists in cell medium supplemented with kininase inhibitors. The endothelin-1 (ET-1) concentration was determined using an Endothelin-1 Quantikine ELISA kit (R&D Systems). Absorbance was measured with a PowerWave microplate reader (BioTek Instruments) at 450 nm and the results were normalized to the protein concentration in samples.

### Statistical analysis

All data from at least three experiments were expressed as mean values ± SD. For statistical comparisons of the mean values in the groups, one-way analysis of variance (ANOVA) was carried out. To show the difference between the groups Tukey post hock test was used. The analysis was performed with the statistical environment R using the "stats" library [27]. The full version of results from the statistical analysis is found in the supporting information (S1 Table).

## Results

### Endogenous expression of bradykinin B2 and dopamine D2 receptors in the HUV-EC-C cell line

**Fig 1:**
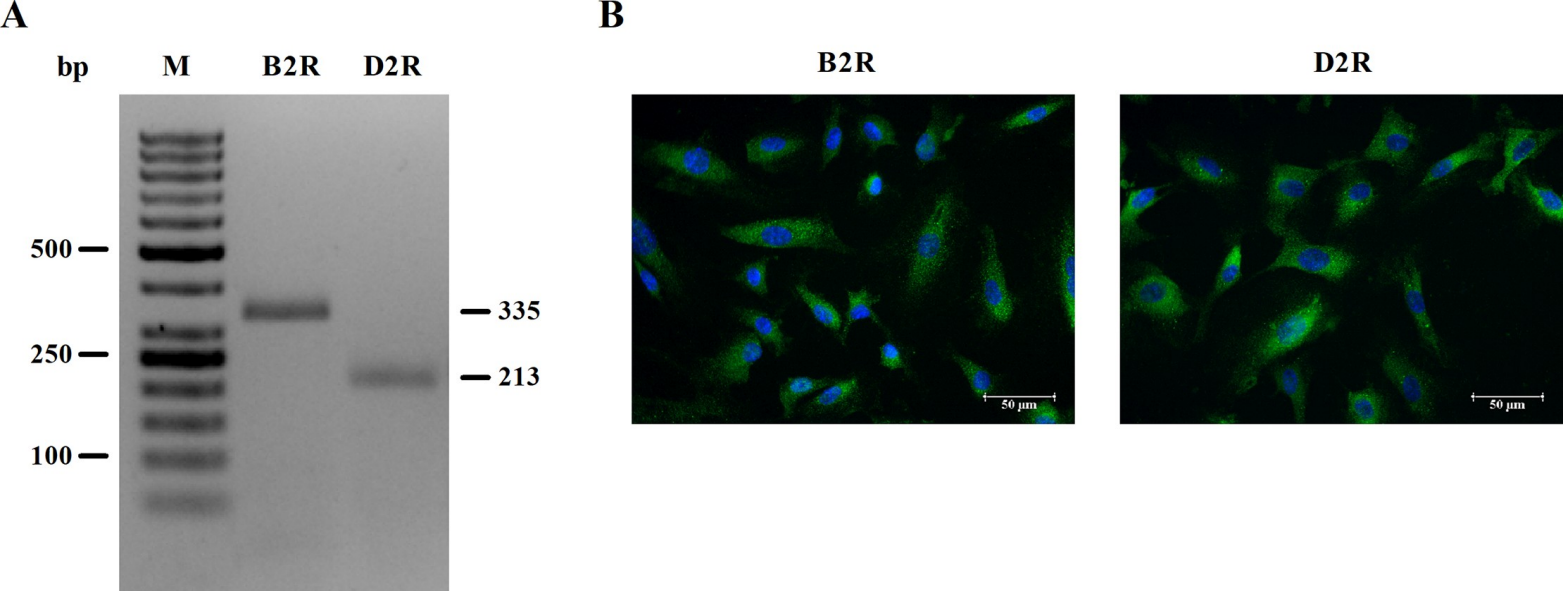
Endogenous expression of B2R and D2R in the HUV-EC-C cell line. (A) The B2R and D2R mRNA levels in cells were detected by RT-PCR. (B) The endogenous expression of B2R and D2R proteins at cell membranes was analyzed by immunocytochemistry using specific antibodies for the extracellular domains of the receptors. The cell nuclei were counterstained with DAPI (blue signal).

To evaluate the effect of the B2R and D2R agonists on the generation of oxidative stress in the endothelial cell line used, we first examined the endogenous expression of both proteins on the surface of these cells. The presence of the two genes was demonstrated by RT-PCR. The PCR products obtained for B2R and D2R, resolved using a 2% agarose gel, were 335 bp and 213 bp, respectively (Fig 1A). In addition, immunocytochemistry studies with specific antibodies against epitopes on the extracellular domains of B2R or D2R in non-permeabilized cells facilitated the detection of these receptors on the cell surface. The resulting images revealed endogenous protein expression of both receptors (green signal) at the cell membrane (Fig 1B). Negative samples were omitted due to lack of a fluorescence signal.

### Effect of B2R and D2R agonists on ROS production

**Fig 2:**
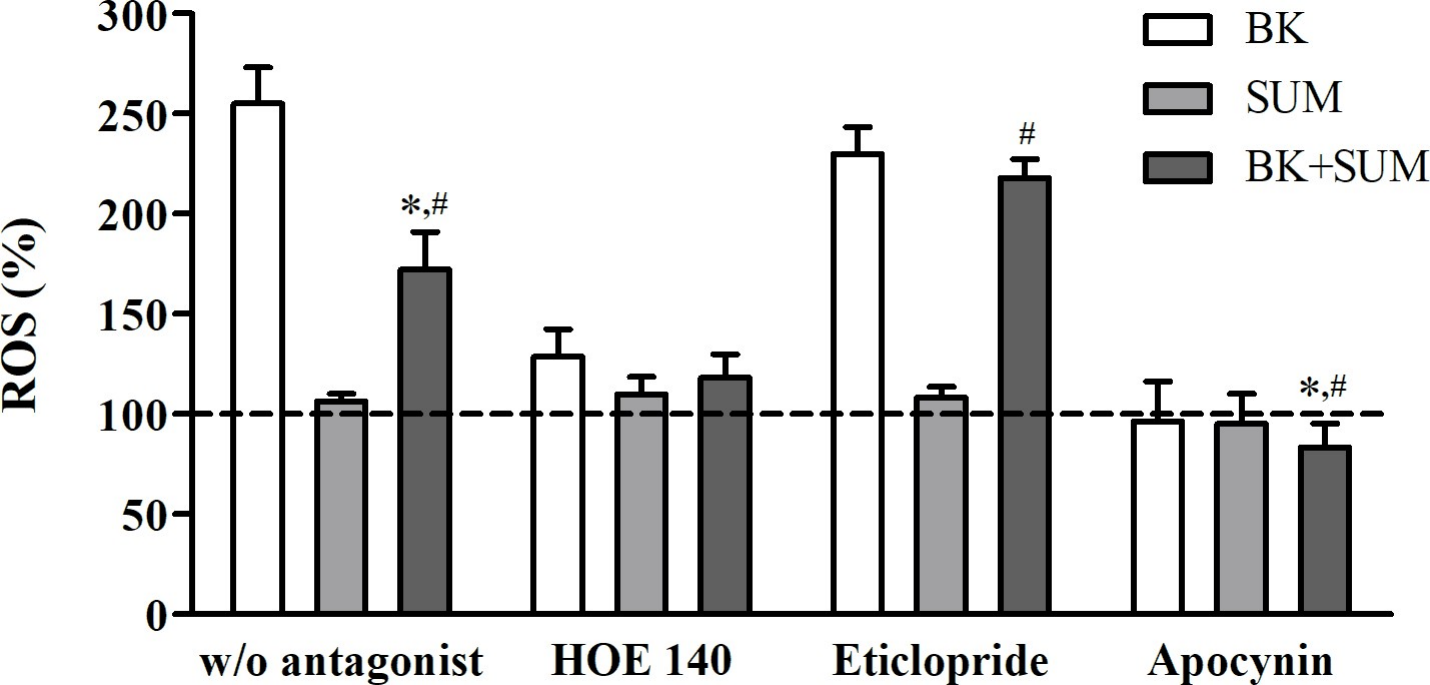
ROS production by HUV-EC-Cs treated with B2R and D2R agonists. DCFH-DA-loaded endothelial cells (1.0 × 10^5^) were stimulated with 100 nM BK, 100 nM SUM, or both agonists simultaneously. Some samples were additionally preincubated with 10 μM HOE 140 or 10 μM eticlopride for 30 minutes or 1 μM apocynin for 1 hour prior to agonist stimulation. The bars represent the mean percentage of the changes in ROS production relative to the untreated cells (with an assumed reference control value of 100%; dashed line). The more significant differences were indicated inside figure. *P < 0.001 *versus* BK-treated cells, ^#^P < 0.001 *versus* SUM-treated cells.

The most commonly used indicator of dysregulated oxidative processes in cells is the ROS level. In this study, changes in ROS generation by endothelial cells were evaluated after treatment with 100 nM BK, considered to be a pathological concentration [28], and/or with SUM, a highly selective D2R agonist, at a concentration above to its EC50 value [29].The results were presented as the percentage of the maximal change in ROS production relative to untreated cells (with an assigned reference value of 100%) (Fig 2). A significant increase in the ROS level was observed after treatment with BK and BK+SUM (by 155% and 72%, respectively) in comparison to the untreated cells. However, the value achieved in samples stimulated with BK+SUM was significantly lower compared with the BK-treated cells (by 83%), indicating a strong diminution of ROS production. Cells incubated with SUM showed no changes in the ROS generation. The preincubation with the B2R antagonist–HOE 140 –resulted in a reduction in ROS production in cells activated with BK and BK+SUM (by 128% and 54%, respectively), compared with the corresponding sample without the antagonist treatment. In turn, prestimulation with eticlopride had no significant effect on the BK action. In this case, the induction of ROS production (by 130%) was similar to that observed in the BK-stimulated sample without the antagonist preincubation. Interestingly, the D2R antagonist was able to invert the effect observed in the BK+SUM-treated cells. ROS production was higher (by 45%) compared with samples stimulated with BK+SUM without the antagonists. The achieved values were also statistically significant (109% increase) compared with the sample treated with SUM after preincubation with eticlopride. HUV-EC-Cs incubated with SUM did not show interesting changes, regardless of whether they were pretreated with the antagonist or not. Prestimulation with apocynin significantly reduced ROS production in the BK- and BK+SUM-treated cells.

### Modulation of antioxidant enzymes in endothelial cells treated with BK and SUM

**Fig 3:**
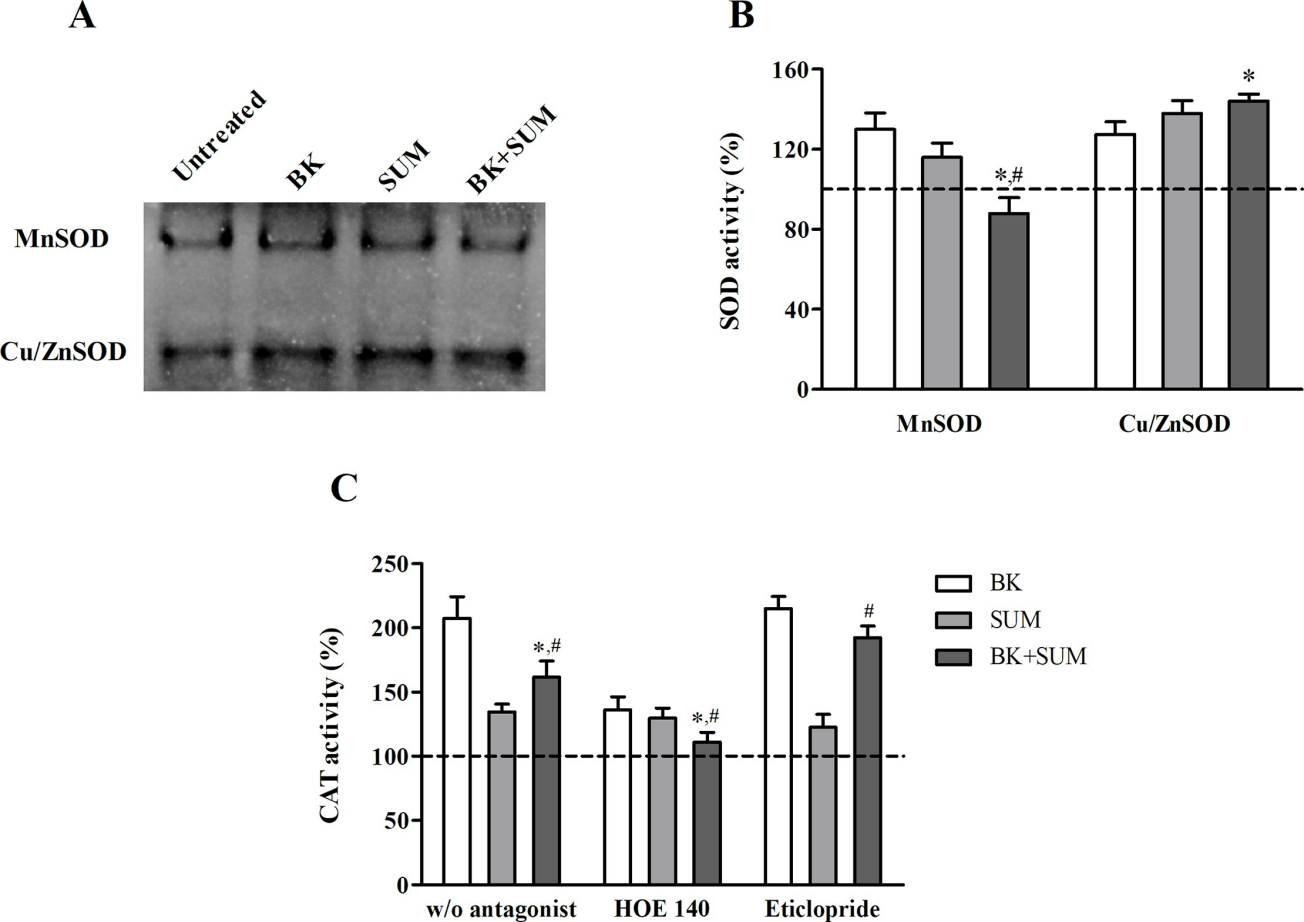
Effect of B2R and D2R agonists on antioxidant enzyme activity. Endothelial cells (1 × 10^6^) were treated with 100 nM BK, 100 nM SUM, or both agonists simultaneously for 2 hours. In some samples, the cells were additionally preincubated with 10 μM HOE 140 or 10 μM eticlopride for 30 minutes prior to receptor agonist stimulation. (A) The enzymatic activity of MnSOD and Cu/ZnSOD in cell lysates normalized to the amount of sample protein was measured after protein separation by native PAGE. (B) The changes in MnSOD and Cu/ZnSOD activity calculated after densitometric gel analysis are presented relative to the untreated cells (with an assumed reference control value of 100%; dashed line). (C) The enzymatic activity of CAT was spectrophotometrically measured and normalized to the amount of sample protein. The figures indicate changes in the percentage of CAT activity relative to the untreated cells (with an assumed reference control value of 100%; dashed line). The bars represent the mean values ± SD from at least three experiments performed in duplicate. *P < 0.001 *versus* BK-treated cells, ^#^P < 0.001 *versus* SUM-treated cells.

The intracellular ROS concentration depends on the action of antioxidant systems. Among others, antioxidant enzymes such as SOD and CAT play a decisive role in regulating the balance between ROS generation and decomposition. Several SOD isoforms regulate oxidative processes–manganese-containing superoxide dismutase mainly in the mitochondria and peroxisomes or copper- and zinc-containing superoxide dismutases, most abundant in the cytoplasm [30]. In our current study, the SOD activity, measured by gel staining after native PAGE, was normalized to the amount of sample protein. The representative gel in grayscale presents the SOD isoforms in the studied samples–the upper band indicates MnSOD and the lower band corresponds to Cu/ZnSOD (Fig 3A). Densitometric analysis of the gel bands allowed a semiquantitative evaluation of the activity of the SOD isoforms (Fig 3B). Stimulation with BK or SUM increased the MnSOD activity by 30% and 15%, respectively, whereas simultaneous treatment with both agonists yielded an opposite result, i.e. showing a slight but significant decrease by 13%. In the case of the Cu/ZnSOD isoform, greater activity was evident in every sample in comparison to the control sample. The obtained values were higher by 27%, 38%, and 44%, respectively, for the BK, SUM, and BK+SUM stimulated cells.

The other antioxidant enzyme investigated in this study was CAT, whose activity was assayed by spectrophotometric monitoring of H_2_O_2_ decomposition and the obtained values were normalized to the sample protein amount. An increase in CAT activity was observed after the treatment with BK, SUM, and BK+SUM (by 107%, 34%, and 61% respectively, Fig 3C). However, the BK+SUM-treated cells showed significantly lower CAT activity compared with the BK-treated and SUM-treated cells. Similar experiments performed in cells that were additionally preincubated with the antagonists showed interesting results. Preincubation with HOE 140 caused a diminution of CAT activity in cells stimulated with BK and BK+SUM (by 70% and 50%, respectively), suggesting specific involvement of B2R, while no changes were observed in the SUM-treated cells, which showed a similar level of activity to the cells without prior exposure to HOE 140 (about a 30% increase). In turn, cell prestimulation with eticlopride produced no effect on the BK action, showing similar CAT activity to that observed in the BK-sample without exposure to this antagonist (about 115%). In addition, eticlopride attenuated the enzyme activity in cells incubated with SUM (by 12%). The most interesting observation was that the BK+SUM-treated cells preincubated with eticloropide showed by 31% higher enzyme activity than in the corresponding sample without the D2R antagonist. This effect was statistically significant compared with the samples treated with BK+SUM with the BK/eticlopride-treated cells.

### Influence of co-treatment with the B2R and D2R agonists on NOS3 activation and NO production in endothelial cells

**Fig 4:**
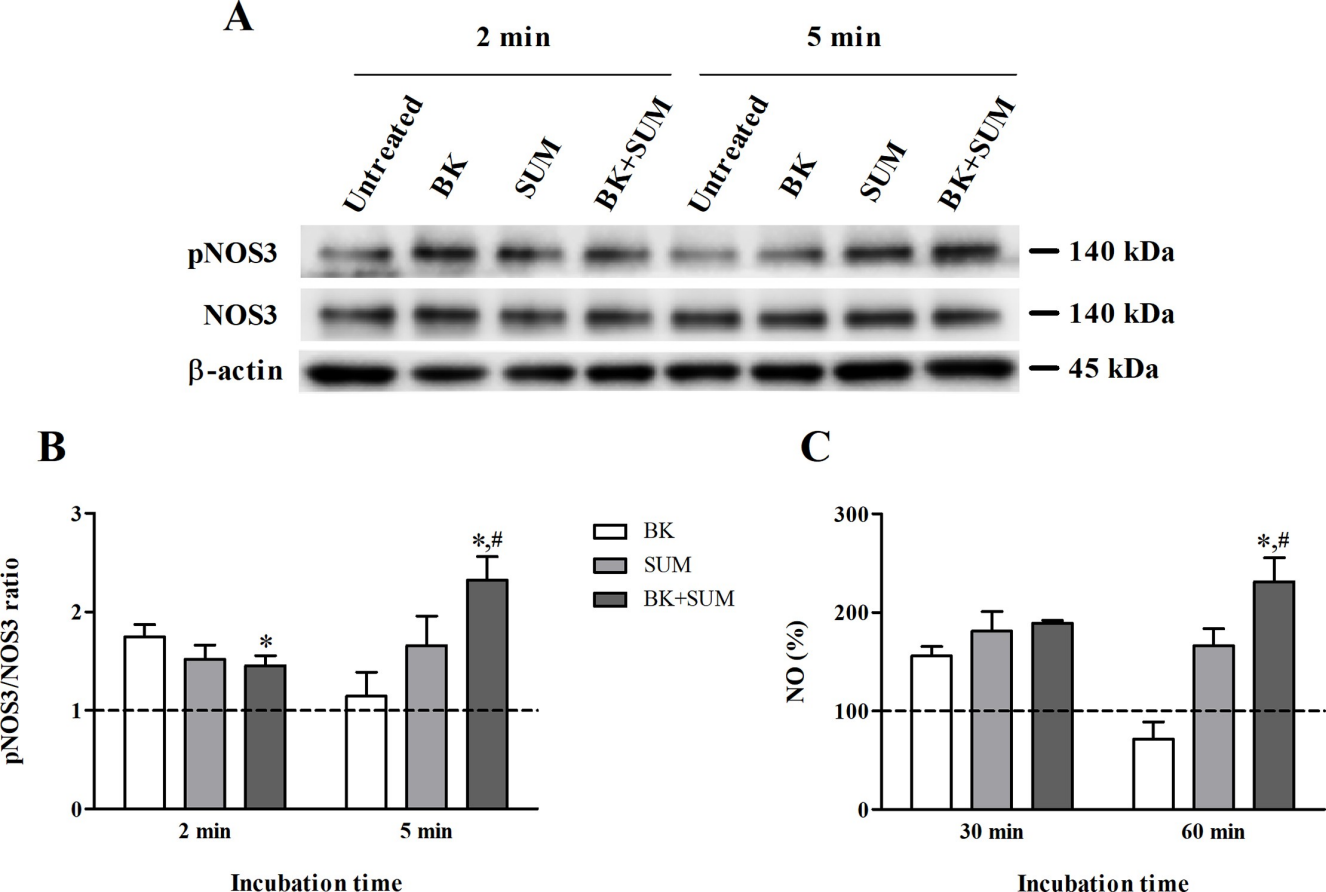
NOS3 activation and NO production by endothelial cells treated with B2R and D2R agonists. Confluent cells were stimulated with 100 nM BK, 100 nM SUM, or both agonists simultaneously for the indicated times. (A) The protein expression levels of pNOS3 Ser1177 and NOS3 were analyzed by western blotting and normalized to the amount of β-actin. (B) The pNOS3/NOS3 ratio was quantified by densitometric analysis. The bars represent the mean values ± SD from three independent experiments as compared to the untreated cells, assumed to have a ratio equal to 1 (dashed line). (C) The percentage of changes in NO production is presented in comparison to the untreated cells, with an assumed reference control value of 100% (dashed line). The mean values ± SD from at least three experiments performed in duplicate are shown. *P < 0.001 *versus* BK-treated cells, ^#^P < 0.001 *versus* SUM-treated cells.

Endothelial nitric oxide synthase is the principal enzyme regulating NO release by endothelial cells. In our study, the activation of NOS3 was examined by measuring the ratio of protein expression of NOS3 phosphorylated at Ser1177 to the total NOS3 protein. Stimulation with BK and SUM at different incubation times was performed and the obtained images are shown in Fig 4A. The pNOS3/NOS3 ratio was calculated after quantification of the corresponding bands by densitometric analysis with Quantity One Software and the obtained values were normalized to β-actin protein expression (Fig 4B). A significant increase in NOS3 phosphorylation was observed in every sample after 2-min stimulation. The highest value was obtained for BK (1.73), followed by SUM (1.51) and BK+SUM (1.45). Longer cell stimulation (5 min) caused a decrease in the pNOS3/NOS3 index in the BK-treated cells (1.14), whereas this value in the SUM-treated sample was similar to that observed after 2 minutes (1.65). However, an interesting effect was observed in the BK and SUM co-stimulated cells, in which a significant increase in NOS3 phosphorylation was achieved, resulting in a pNOS3/NOS3 ratio of 2.32.

The analysis of NOS3 activation was supplemented by additional measurements of NO release by endothelial cells. NO production was measured by assaying the nitrite concentration in cell supernatants with a fluorometric technique with an assigned reference value of 100% for untreated cells. The obtained results (Fig 4C) revealed time-dependent changes in NO release after stimulation with the receptor agonists. The BK-treatment increased the NO concentration by 55% after 30 minutes, whereas longer incubation for 1 hour led to its decrease by about 30%. The most remarkable observations were made in the BK+SUM samples, in which 30-min stimulation caused a significant increase in the NO concentration (by 80%) and longer incubation increased it even by 131%. The SUM-treated cells also showed enhanced NO release (by 80% and 100% for 30-min and 60-min incubation, respectively).

### Inflammatory response of endothelial cells induced by the B2R and D2R agonists

**Fig 5:**
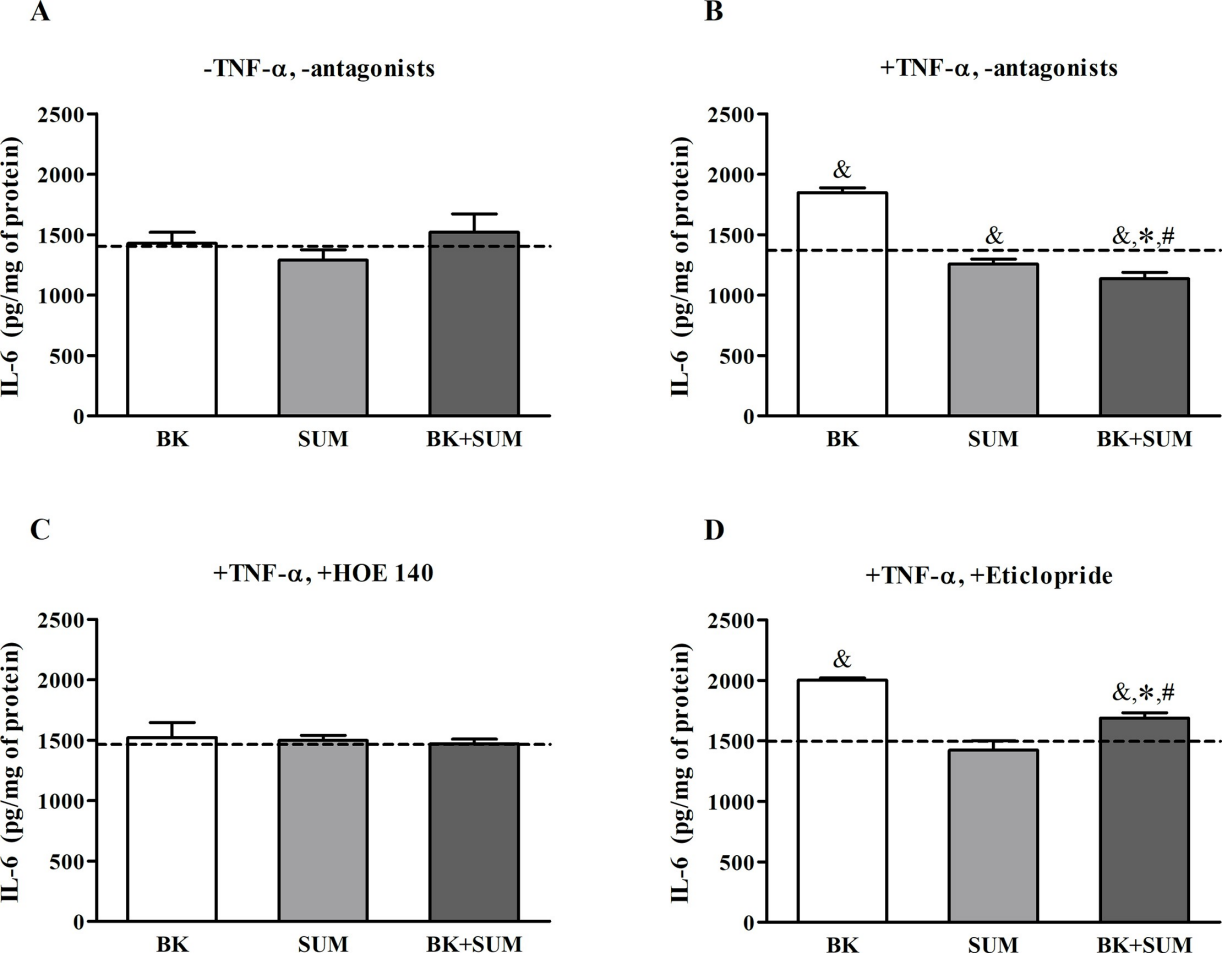
IL-6 release by endothelial cells stimulated with B2R and D2R agonists. Confluent cells, (A) preincubated or (B) non-preincubated with 10 ng/ml TNF-α for 24 hours, were stimulated with 100 nM BK, 100 nM SUM, or both agonists simultaneously for 6 hours. Some samples were additionally pretreated with (C) 10 μM HOE 140 or (D) 10 μM eticlopride for 30 minutes prior to receptor agonist induction. The protein production of IL-6 in the culture medium was analyzed by ELISA and normalized to the amount of sample protein. The bars present the mean values ± SD from at least three experiments in triplicate. ^&^P < 0.001 *versus* untreated cells, *P < 0.001 *versus* BK-treated cells, ^#^P < 0.001 *versus* SUM-treated cells.

Cell responses that are dependent on oxidative stress processes are associated with the production of a variety of proinflammatory factors. We here analyzed the role of the B2R and D2R agonists in regulating the release of IL-6, i.e. one of the first cytokines appearing during inflammatory response. Cells without TNF-α pretreatment did not show any significant changes in IL-6 production after the receptor agonist treatment (Fig 5A). In turn, an increase in IL-6 release was registered in the TNF-α-stimulated cells after incubation with BK (by 34%), whereas a weak decrease was observed in the SUM-treated cells (Fig 5B). However, the simultaneous stimulation with BK and SUM led to the total abolition of the BK-induced effect, showing even a significant decrease in IL-6 release compared to the untreated cells (by 18%). To confirm the effects of the receptor agonists, experiments were also performed in cells pretreated with specific antagonists. The use of HOE 140 reversed the achieved effect on IL-6 production in the BK- and BK+SUM-treated cells and had no influence on the SUM-stimulated cells. In the latter sample, the IL-6 concentration was comparable to the value observed in the untreated cells (Fig 5C). In turn, eticlopride effectively inhibited the effect observed in cells treated with BK+SUM, whereas no changes were produced by this antagonist in the BK- or SUM-stimulated cells (Fig 5D). In the case of the BK+SUM sample, a significant increase in IL-6 production was observed (by 13%, relative to cells treated only with eticlopride). It should also be noted that used receptor antagonists did not have any significant influence on the cytokine release by endothelial cells. The IL-6 concentration was unaffected in the untreated cells regardless of any preincubation with HOE 140 or eticlopride (1442.6 pg/mg of protein).

### Influence of BK and SUM on the expression of pro- and anti-apoptotic proteins in endothelial cells

**Fig 6:**
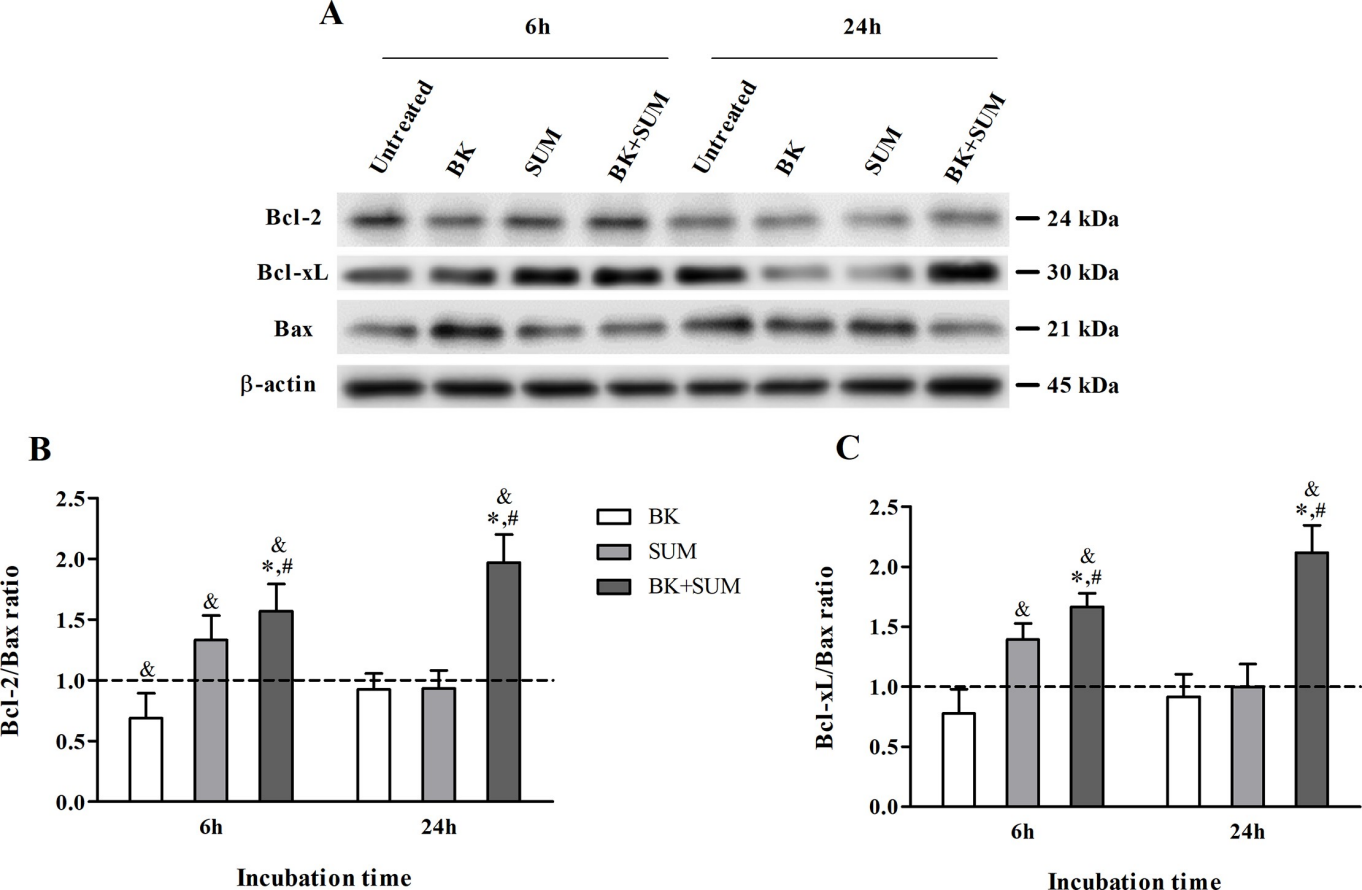
Effect of B2R and D2R agonists on the expression of pro- and anti-apoptotic proteins. Cells (1.0 × 10^6^) were treated with 100 nM BK, 100 nM SUM, or both agonists simultaneously for 6 or 24 hours. (A) The protein expression levels of Bcl-2, Bcl-xL, and Bax was analyzed by western blotting. The (B) Bcl-2/Bax and (C) Bcl-xL/Bax ratios were calculated by densitometric analysis and the values were normalized to the β-actin expression level. The figures represent the mean values ± SD from two experiments, compared with the value obtained for the untreated cells, assumed to be 1 (dashed line). ^&^P < 0.001 *versus* untreated cells, * P < 0.001 *versus* BK-treated cells, ^#^P < 0.005 *versus* SUM-treated cells.

An impaired oxidant-antioxidant balance and enhanced inflammatory responses can lead to the development of apoptotic processes. We examined the effect of BK and SUM on the expression of the pro-apoptotic protein Bax and the anti-apoptotic proteins Bcl-2 and Bcl-xL by western blotting. The detected bands for these proteins indicated significant changes in their expression in endothelial cells (Fig 6A). The Bcl-2/Bax (Fig 6B) and Bcl-xL/Bax (Fig 6C) ratios were calculated by densitometric analysis of the corresponding bands normalized to the β-actin protein content. The ratio values were compared to that for untreated cells, which were given a reference value of 1. Both Bcl-2/Bax and Bcl-xL/Bax ratios showed a similar effect depending of the incubation time, presenting an insignificant decrease in cells stimulated with BK for 6 hours (0.68 and 0.77, respectively). In contrast, cells incubated with SUM and with BK+SUM showed higher values of both indexes compared to the untreated cells. The Bcl-2/Bax ratio increased to 1.33 and 1.56 in the SUM- and BK+SUM-treated cells, respectively. In the case of the Bcl-xL/Bax ratio, the values increased by 0.39 and 0.66, accordingly. Extended stimulation caused an increasing tendency toward an anti-apoptotic response only in cells treated with both agonists. After 24 hours, the calculated Bcl-2/Bax and Bcl-xL/Bax indexes were 1.96 and 2.11, respectively. Moreover, no significant changes were observed in cells stimulated with BK or SUM alone during a 24-hour period, as the ratio values were comparable to those in the untreated samples.

### Caspase 3/7 activity in endothelial cells treated with B2R and D2R agonists

**Fig 7:**
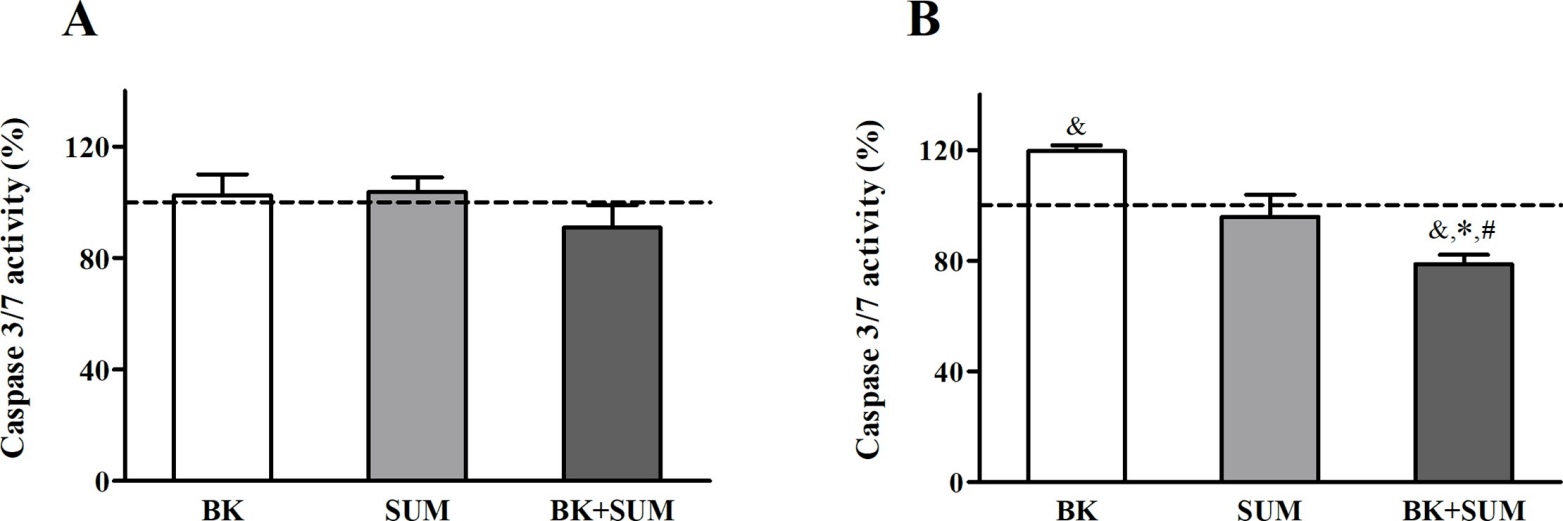
Relative caspase 3/7 activity in endothelial cells after treatment with B2R and D2R agonists. Cells (1.0 × 10^4^) without (A) preincubation or (B) preincubated with 10 ng/ml TNF-α for 6 hours were treated with 100 nM BK, 100 nM SUM, or both agonists simultaneously for 24 hours. Enzyme activity was analyzed with a chemiluminescent assay. The figures present the mean values ± SD of the percentage change in caspase 3/7 activity compared with the untreated cells (with an assumed reference control value of 100%; dashed line). The results were obtained from at least three experiments performed in triplicate. ^&^P < 0.001 *versus* untreated cells, *P < 0.001 *versus* BK-treated cells, ^#^P < 0.001 *versus* SUM-treated cells.

Changes in the expression of Bax, Bcl-2, and Bcl-xL proteins are commonly associated with the modulation of the caspase cascade. We thus assessed the influence of the B2R and D2R agonists on the caspase activity, which is induced by pro-apoptotic stimuli. Cells treated with BK, SUM, or both agonists simultaneously showed no significant changes in caspase 3/7 activity (Fig 7A). Nonetheless, cells preincubated with the proinflammatory cytokine TNF-α demonstrated an interesting profile of caspase activity under the treatment with the receptor agonists (Fig 7B). Increased enzyme activity was observed in the samples treated with BK alone (by 20%), but was significantly reduced by 22% after exposure to both BK and SUM, with no significant differences in the cells stimulated with SUM alone.

### Endothelin-1 release by endothelial cells by the B2R and D2R agonists

**Fig 8:**
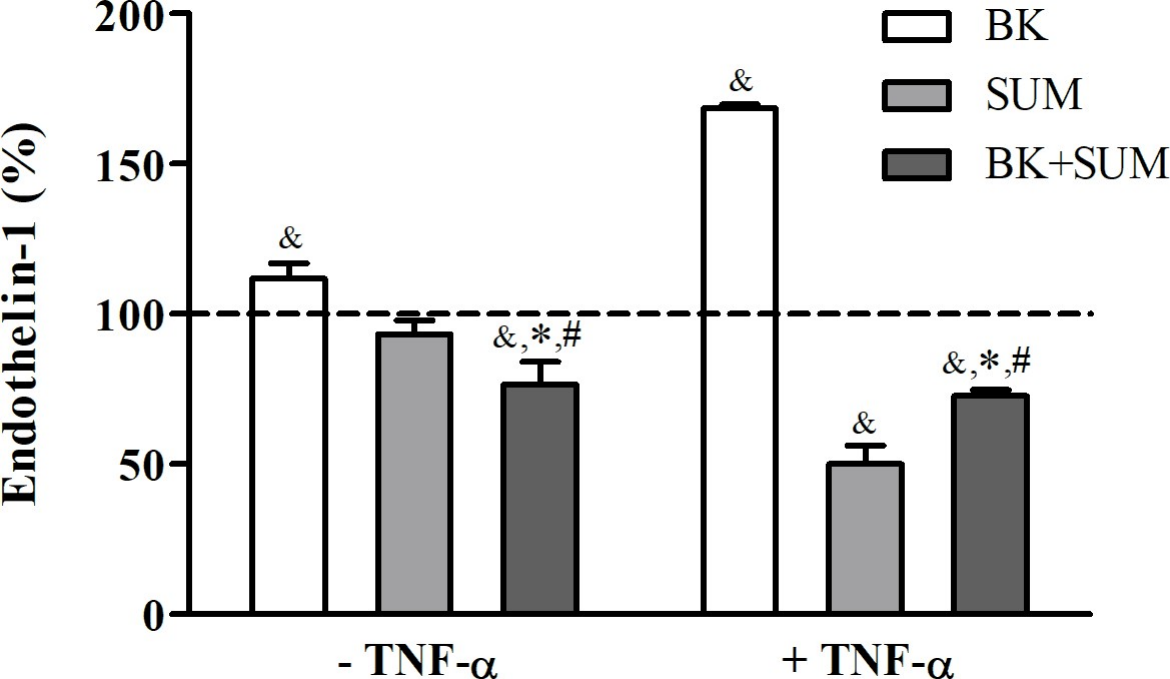
Endothelin-1 release by endothelial cells stimulated with B2R and D2R agonists. Confluent cell cultures were preincubated with 10 ng/ml TNF-α for 6 hours, followed by stimulation with 100 nM BK, 100 nM SUM, or both agonists simultaneously for 24 hours and the ET-1 concentration was measured by ELISA. The bars present the mean values ± SD of the percentage change in endothelin-1 production compared with the untreated cells (with an assumed reference control value of 100%; dashed line). The results were obtained from at least three experiments performed in duplicate. ^&^P < 0.001 *versus* untreated cells, *P < 0.001 *versus* BK-treated cells, ^#^P < 0.001 *versus* SUM-treated cells.

Endothelin-1, i.e. an endothelium-derived vasoactive peptide, is involved in promotion of cytokine and ROS generation [5]. In our study, a slight but significant increase in ET-1 release was registered in cells stimulated with BK (by 11%), whereas a weak decrease was observed in the SUM-treated cells. On the other hand, the simultaneous stimulation with BK and SUM led to the total abolition of the effect exerted by the BK treatment (Fig 8A), finally with even a significant decrease in ET-1 release (by 24% compared to untreated cells). Moreover, endothelial cells preincubated with TNF-α showed stronger changes in the ET-1 profile after the treatment with the receptor agonists. In samples treated only with BK, the ET-1 release was significantly increased (by 68%), while cells treated with SUM or simultaneously with BK and SUM showed reduced ET-1 production in comparison to the control samples (by 50% and 28%, respectively).

## Discussion

Numerous studies to date have confirmed that endothelial dysfunctions are a hallmark of a wide range of cardiovascular diseases, such as atherosclerosis and hypertension, which are also associated with oxidative stress and an inflammatory state [31]. Based on our prior study, which demonstrated an interaction between these two receptors with alterations of their signaling pathways [22], it could be speculated that such interaction would modify any agonist-induced effects on endothelial functions. Taking into consideration that a high BK concentration in the blood may develop inflammatory processes in the vascular system [5–6], the goal of our current study was to define a possible role of the cooperation between B2 and D2 receptors in regulating processes related to oxidative stress, inflammation, and apoptosis in endothelial cells. These studies seem to be interesting, especially taking into account previous reports indicating anti-oxidative effects exerted by D2R activation [14]. In accordance with the convincing evidence to date that B2R and D2R are endogenously expressed in primary endothelial cells isolated from the umbilical vein [18, 32], we have confirmed here that these proteins are abundantly expressed in the endothelial cell line used in this study (Fig 1).

The imbalance between the production and decomposition of ROS, which normally act as secondary cellular messengers, leads to the state known as oxidative stress. In the present study, BK-induced ROS overproduction was detected and found to be nearly completely inhibited by HOE 140 (Fig 2), indicating that this effect was mainly related to B2R activation. In contrast, the eticlopride pretreatment had no impact on the BK-induced response. Unlike our present observations, previous reports have suggested a protective action of BK against ROS production in endothelial cells [8, 33]. It should be pointed out, however, that endothelial cells were treated with a BK concentration near to the physiological level in those studies, whereas we used a higher dose and additional supplementation with kininase inhibitors to avoid rapid BK degradation [28]. On the other hand, some studies using BK at concentrations of 1–10 μM reported enhanced production of several reactive oxygen species in different endothelial cells via the NADPH oxidase-dependent pathway [10–12]. Consequently, our results have confirmed that the BK-induced effect in the HUV-EC-C cell line may provide a pathological endothelial cellular model. The most relevant observations from the present analyses were related to the fact that the cell stimulation with the D2R agonist did not alter the basal ROS level, whereas co-stimulation with BK and SUM significantly diminished the BK-induced effect on ROS production. Furthermore, eticlopride reversed this effect, suggesting that it occurs via a direct role of D2R, changing the B2R signaling. Hence, we demonstrate here that the selective D2R agonist sumanirole can counteract the pathological effects induced by BK. Moreover, apocynin abolished the effects exerted by the receptor agonists, indicating the exclusive role of NADPH oxidase in these responses. This enzyme is considered to be the predominant source of ROS in the endothelium [12].

The intracellular ROS concentration depends not only on their production but also on their subsequent metabolism by antioxidant enzymes, such as SOD and CAT. SOD is the principal defense against superoxide radicals in cells by catalyzing their dismutation to oxygen and hydrogen peroxide. Since we demonstrated the regulation of ROS production by the B2R and D2R agonists, we subsequently investigated whether the agonists could also modulate the activity of antioxidant enzymes. The role of BK in the upregulation of SOD activity was indicated previously in cardiomyocytes pretreated with H_2_O_2_ as well as *in vivo* in hyperglycemic rats [9, 34]. It was also demonstrated that D2R agonists, such as ropinirole and bromocriptine, similarly cause up-regulation of SOD activity in mice striatum and cardiomyocytes [16, 35]. In accordance with these observations, our current study also showed the induction of both SOD isoforms in endothelial cells under the action of BK and SUM. Surprisingly, the co-stimulation with both agonists gave unexpected results (Fig 3B), showing a significant decrease in MnSOD activity in comparison to the Cu/ZnSOD isoforms. This difference between SOD isoform activities was quite interesting because the half-life of MnSOD is relatively long, compared to the mainly cytosolic Cu/ZnSOD [36]. However, we observed reduced ROS release in response to the co-stimulation with BK and SUM, which correlates with the declining MnSOD activity, suggesting a significantly lower demand for this enzyme under these conditions. Another alternative explanation may be related to intensive use of this enzyme in the destruction of superoxide radicals, which leads to a decrease in SOD concentration and consequently in enzyme activity. Therefore, taking into account the fact that the production of superoxide occurs primarily in mitochondria [5], the observed low activity of MnSOD after the co-treatment with both agonists may be attributed to the antioxidative effect caused by the B2R-D2R interaction.

As mentioned previously, the action of SOD leads to an enhanced production of another component of ROS, hydrogen peroxide. Cells are protected from the negative effects of H_2_O_2_ by CAT, which rapidly decomposes H_2_O_2_ to water and oxygen [30]. In this study, we demonstrated a significant BK-induced increase in CAT activity, an effect that was partially inhibited by HOE 140, confirming the participation of B2R (Fig 3C). A previous report also showed the participation of BK in CAT induction [34]. Thus, we suggest that B2R activation in endothelial cells leads to rapid ROS release, including superoxide radicals and hydrogen peroxide, which are scavenged by SOD and CAT to maintain the balance between pro- and antioxidative systems. On the other hand, the role of ropinirole, i.e. a D2R agonist, in CAT activation to regulate oxidative stress was previously confirmed in the mouse striatum [16]. We did not observe significant changes in CAT activity after the cell incubation with SUM. Nevertheless, the effect resulting from the BK+SUM stimulation in our study was noteworthy. Simultaneous treatment with both agonists showed a strong decrease in the BK-induced CAT activity, which was reversed by the cell preincubation with eticlopride. These observations suggest a cooperative action of B2R and D2R in the regulation of oxidative stress in endothelial cells. Interestingly, the BK+SUM effects on CAT activity in endothelial cells correlated with ROS production (Fig 2). Similar as in the case of decreased SOD activity we can assume that the impaired CAT activity may be attributed to a lower demand of the enzyme because the production of ROS is diminished. Ultimately, our findings suggest that the cooperation between B2R and D2R not only downregulates ROS production in endothelial cells but also alters antioxidant systems, responsible for the maintenance of the balance between pro- and antioxidative processes.

Nitric oxide is a pivotal regulator of endothelial functions, e.g. vascular tone. In endothelial cells, NO is produced by the NOS3 isoform after its activation by intracellular Ca^2+^ ions and phosphorylation at Ser1177 [37]. Reduced NO production is mostly associated with cardiovascular diseases [38]. One of the main causes of altered NO release by cells is the inhibition of NOS3 phosphorylation. Our results (Fig 4B) are in accord with a previous study on endothelial cells, in which stimulation with 1 μM BK led to a rapid induction of NOS3 phosphorylation [39]. However, after 5-min incubation, this effect was attenuated. Based on the fact that SUM produced only a weak increase, irrespective of the incubation time, the strong NOS3 activation observed in cells stimulated with BK + SUM after 5 minutes can be attributed mainly to a cooperation between the B2R and D2R receptors. It is possible that these receptors compete for G proteins, resulting in modified receptor signaling. B2R regulates calmodulin binding to NOS3 in a Ca^2+^-dependent manner, causing enzyme activation and NO production, whereas D2R can activate NOS3 via a cAMP-dependent pathway [37, 40]. Hence, signaling changes induced during endothelial cell co-stimulation with BK and SUM cannot be excluded. Indeed, during late D2R signaling, a different cAMP-independent pathway for Akt activation is also proposed [41], which is required for NOS3 phosphorylation [37]. Therefore, additional detailed studies on B2R and D2R signaling are desirable.

The measurements of nitrite, i.e. one of terminal NO metabolites, were consistent with the observed NOS3 phosphorylation levels. A transient rise in the NO release by BK was observed, with the highest peak seen after 30 minutes (Fig 4C). Similar results were previously obtained by Cokić and co-workers, who reported a quick and transient endothelial cell response pattern that is dependent on the NO induced strictly by B2R activation [42]. On the contrary, the D2R involvement in the regulation of NO production has not been well studied. Thus far, there has been some evidence indicating a specific role of this receptor in inducing NOS3 expression in vascular smooth muscle and endothelial cells but NO release was not measured [40]. We here report for the first time the involvement of D2R not only in NOS3 phosphorylation but also in NO production by endothelial cells. Interestingly, in comparison to BK, the SUM-dependent response was consistent and longer, as the BK-induced effect was strongly reduced after 1-hour incubation. Simultaneous treatment of HUV-EC-Cs with both agonists led to a growing increase in nitric oxide production, which depended on the incubation time. It should be noted that these effects correlate with the measured levels of NOS3 activation. These findings confirm that D2R agonists can regulate BK activities through the modulation of cellular functions dependent on NO production. It is worth mentioning that increased NO scavenging by ROS has been associated with reduced NO bioavailability and, therefore, with impaired endothelium functionality, including the development of oxidative stress [5]. Hence, augmented NO production by BK+SUM-treated cells may prevent the early development of oxidative stress. However, it should be noted that NO production sometimes can lead to an increased production of peroxynitrite radicals, especially when antioxidant systems do not work properly.

Several studies affirm that many proinflammatory cytokines are triggered by oxidative stress, especially by mitochondrial ROS [31]. Increased release of intracellular ROS leads to the stimulation of an important proinflammatory factor NF-κB, which plays a pivotal role in the induction of cytokine expression, particularly IL-6 [43]. We did not observe any significant effect on IL-6 production following the endothelial cell stimulation with the B2R or D2R agonists. However, the preincubation of HUV-EC-Cs with TNF-α, which to some extent reflects a model of inflamed cells, allowed detection of significant changes in cytokine release (Fig 5). In brief, SUM was incapable of prompt IL-6 production, whereas the BK-induced cytokine release was blocked when the cells were co-incubated with both agonists. The use of the receptor antagonists confirmed the mediation of B2R and D2R in the observed effects. These results agree with a previous report on the regulation of IL-6 production by BK [44]. Nevertheless, our study provides new observations regarding the D2R-mediated regulation of the inflammatory response induced by BK. In fact, the anti-inflammatory effects of D2R were described in a recent report [45] showing reduced expression of the NF-κB protein in a mouse experimental model of intracerebral hemorrhage. In total, it seems that the high degree of NOS3 phosphorylation after the BK+SUM stimulation with subsequent enhanced NO release could be helpful in the diminution of the IL-6 concentration in the cell culture medium, especially considering a previous report demonstrating that a higher cytokine concentration correlates with the inhibition of NOS3 phosphorylation at Ser1177 [46].

Cell apoptosis can be initiated by several mechanisms, including intrinsic signals such as oxidative stress. The sensitivity of cells to apoptotic stimuli depends on the balance between anti-apoptotic (e.g. Bcl-2 and Bcl-xL) and pro-apoptotic proteins (e.g. Bax). The interactions between these two groups of proteins determine the initiation of the caspase cascade, including caspase 3, an important effector molecule in apoptosis [47]. Taking this into account, we investigated whether the cooperation between B2R and D2R may result in the regulation of apoptotic processes in endothelial cells. A decisive pro-apoptotic effect of BK has not been demonstrated. However, Oeseburg and co-workers reported attenuation of H_2_O_2_-induced apoptosis in bovine aortic endothelial cells under the action of BK at a physiological concentration [33]. Similar results were obtained in other studies performed in pig cerebral microvascular endothelial cells stimulated with H_2_O_2_ or lipopolysaccharide and in serum-deprived HUVEC cells [48–49]. This study has revealed a weak pro-apoptotic effect induced by BK at a pathological concentration in endothelial cells (Fig 6). The ratio of anti- to pro-apoptotic proteins decreased after 6-h incubation, whereas longer incubation with BK did not exert any notable effect. Based on these results, we could not conclude that BK promotes or attenuates apoptosis, especially as no changes were observed in caspase 3/7 activity in the BK-treated cells (Fig 7A). It seems therefore that this peptide alone is not able to induce apoptotic pathways in the endothelium. However, when the cells were additionally pretreated with the proinflammatory cytokine TNF-α, a weak but significant increase in caspase 3/7 activity was induced (Fig 7B). Interestingly, a similar result was observed during our analysis of IL-6 production (Fig 5), confirming that, upon stress and proinflammatory stimuli, high doses of BK can potentiate not only endothelium dysfunctions but also pro-apoptotic effects. On the other hand, our findings indicate a slight increase in the anti-apoptotic ratio after 6-h incubation with SUM (Fig 6A, 6B and 6C). Different reports provide evidence showing the participation of bromocriptine, i.e. a D2R agonist, in the down-regulation of caspase 3 activity and the induction of Bcl-2 protein expression in rat cardiomyocytes after myocardial ischemia [35, 50]. Moreover, Nair and co-workers [51] showed the inhibition of H_2_O_2_-induced apoptotic processes by bromocriptine in nigral dopamine and PC-12 cell lines. However, our study showed that caspase 3/7 activity was unchanged, indicating no anti-apoptotic effects of the D2R action (Fig 7). These results suggest that SUM alone plays no significant role in the control of apoptosis in endothelial cells. This clear discrepancy with previous reports may be related to the specificity of the D2R agonists in these studies. Bromocriptine possesses a higher binding affinity for dopamine receptor type 3 than for D2R, whilst SUM is a highly selective agonist for D2R [29]. Nevertheless, the surprising results obtained when the cells were treated simultaneously with both agonists argue for an anti-apoptotic role of D2R. The level of pro-apoptotic proteins (Fig 6) as well as the activity of caspase 3/7 in the TNF-α–pretreated endothelial cells after stimulation with both BK and SUM was significantly reduced (Fig 7B). These results strongly indicate a protective effect on endothelial cells exerted by the cooperation of B2R and D2R, which also prevents the development of apoptosis. The anti-apoptotic action of the B2R/D2R agonists may also be related to the pNOS3-dependent pathway and NO production. The inhibition of apoptosis by NO in different cell types, including endothelial cells, has been demonstrated [47]. Moreover, the prevention of apoptosis has been reported to correlate mainly with a reduced calcium overload [35], and we recently reported the abolition of BK-induced calcium release in HEK293 cells treated simultaneously with B2R and D2R agonists [22]. Nevertheless, our studies were performed in cellular model of endothelium, in which the observed changes in apoptotic indicators no necessarily may lead to apoptosis. To obtain a more reliable response about BK and SUM action in apoptosis it is necessary to introduce a more sophisticated model, for example in vivo studies.

On the other hand, secretion of endothelin-1, a vasoactive peptide involved in the pathophysiology of many cardiovascular diseases such as hypertension, atherosclerosis, and hypercholesterolemia, is dependent on the intracellular calcium concentration [52]. Increased ET-1 concentration in blood is a positive indicator of endothelium dysfunction. Previous reports have indicated BK mediation in the induction of ET-1 release [52–53]. Our study provides results that confirm this observation (Fig 8) and, in addition, reports for a first time a protective role of the D2R agonist in the regression of the BK-dependent production of endothelin-1. Therefore, the effect of SUM on the regulation of endothelial cell dysfunction induced by BK confirms our hypothesis that both receptors may interact and regulate cellular function. We suppose that the conformational changes of receptors’ structure after dimerization may hinder or facilitate the access to one of the agonists or maybe the receptors compete with each other to the appropriate G protein. Of course, we cannot exclude that other GPCRs, which can also dimerize with B2R and/or D2R, or can at least partly contribute to the observed effects. However additional studies including analysis in vivo or ex vivo could provide more reliable information.

**Fig 9:**
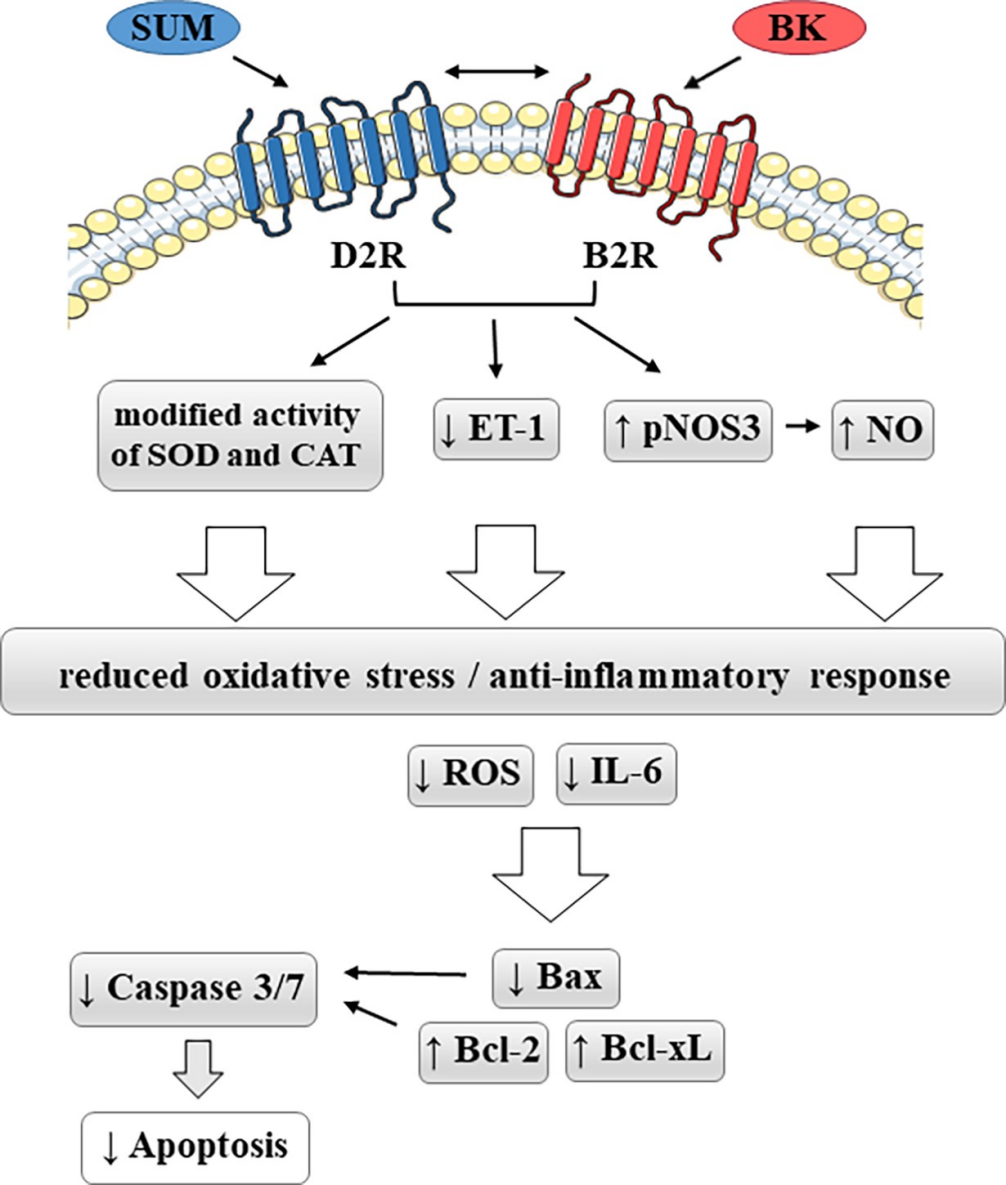
Involvement of B2R and D2R in oxidative and inflammatory responses as well as in apoptotic processes in endothelial cells. BK–bradykinin, B2R –bradykinin B2 receptor, CAT–catalase, D2R- dopamine D2 receptor, ET-1 –endothelin-1, IL-6 –interleukin-6, MnSOD–mitochondrial superoxide dismutase, pNOS3 –phosphorylated endothelial nitric oxide synthase, ROS–reactive oxygen species, SUM–sumanirole. Thick arrows represent mechanisms that may not necessarily occur directly and in which additional factors may be involved.

In conclusion, our data indicate a significant role of D2R in the regulation of the effects induced by a B2R agonist on oxidative stress, and on inflammatory as well as apoptotic processes in endothelial cells (Fig 9). These changes seem to be associated with the cooperation between these receptors, possibly during direct interactions at the cell membrane, which can modify their typical signaling pathways. Taking into consideration that the imbalance of the aforementioned processes leads to endothelial dysfunction, its regulation could probably protect or even reverse vascular pathologies. Thus, the findings presented herein enrich the existing knowledge of the complex functioning of the endothelium, which may open up new perspectives in the search for modulators of B2 and D2 receptors to develop new drug candidates with potential therapeutic applications in the treatment of cardiovascular disorders.

## Supporting information

S1 Table Statistical analysis of the results. Comparison of mean values in the groups was performed with one-way analysis of variance (ANOVA). To show the difference between the groups Tukey post hock test was used.
(PDF)

S2 Table Original results. Normalized original results from at least three experiments are presented in separate tables.
(PDF)

## Abbreviations

BK: bradykinin
B2R: bradykinin B2 receptor
CAT: catalase
D2R: dopamine D2 receptor
ET-1: endothelin-1
GPCRs: G protein-coupled receptors
HOE 140: icatibant
IL-6: interleukin-6
NOS3: endothelial nitric oxide synthase
ROS: reactive oxygen species
SOD: superoxide dismutase
SUM: sumanirole
TNF-α: tumor necrosis factor alpha
