# Supplementary material for: Influence of bradykinin B2 receptor and dopamine D2 receptor on the oxidative stress, inflammatory response, and apoptotic process in human endothelial cells
Source: PLoS One. 2018 Nov 14;13(11):e0206443. doi: 10.1371/journal.pone.0206443 (PMC6241119; doi:10.1371/journal.pone.0206443)
Supplement: S1 Table — Comparison of mean values in the groups was performed with one-way analysis of variance (ANOVA). To show the difference between the groups Tukey post hock test was used. (PDF) [file pone.0206443.s001.pdf]

**ROS**

| | Anova | | Tukey - posthock test | |
|---|---|---|---|---|
| | groups | *p-value* | compared gropus | *p-value* |
| Without_antagonists | BK<br>SUM<br>BK+SUM<br>Untreated | <2e-16 | BK-Untreated | 8.251178e-13 |
| | | | SUM-Untreated | 8.973312e-01 |
| | | | BK_SUM-Untreated | 1.166110e-07 |
| | | | SUM-BK | 8.267831e-13 |
| | | | BK_SUM-BK | 1.111311e-08 |
| | | | BK_SUM-SUM | 1.232118e-06 |
| With_HOE_140 | BK<br>SUM<br>BK+SUM<br>Untreated | 6.92e-07 | BK-Untreated | 5.223396e-07 |
| | | | SUM-Untreated | 1.471513e-01 |
| | | | BK_SUM-Untreated | 6.211034e-04 |
| | | | SUM-BK | 6.665371e-04 |
| | | | BK_SUM-BK | 1.538416e-01 |
| | | | BK_SUM-SUM | 1.774635e-01 |
| With _Eticlopride | BK<br>SUM<br>BK+SUM<br>Untreated | <2e-16 | BK-Untreated | 8.248957e-13 |
| | | | SUM-Untreated | 5.234461e-01 |
| | | | BK_SUM-Untreated | 8.265610e-13 |
| | | | SUM-BK | 8.266721e-13 |
| | | | BK_SUM-BK | 5.330547e-01 |
| | | | BK_SUM-SUM | 8.398837e-13 |
| With _Apocynin | BK<br>SUM<br>BK+SUM<br>Untreated | 2.98e-05 | BK-Untreated | 5.605618e-01 |
| | | | SUM-Untreated | 8.298937e-01 |
| | | | BK_SUM-Untreated | 3.701274e-05 |
| | | | SUM-BK | 9.670915e-01 |
| | | | BK_SUM-BK | 2.320818e-03 |
| | | | BK_SUM-SUM | 5.705885e-04 |

| | | Anova | | **Tukey - posthock test** | |
|---|---|---|---|---|---|
| | | groups | *p-value* | compared gropus | *p-value* |
| **SOD** | MnSOD | BK<br>SUM<br>BK+SUM<br>Untreated | 5.77e-12 | BK-Untreated | 3.196108e-09 |
| | | | | SUM-Untreated | 3.572128e-05 |
| | | | | BK_SUM-Untreated | 9.253939e-04 |
| | | | | SUM-BK | 3.550626e-04 |
| | | | | BK_SUM-BK | 6.409873e-12 |
| | | | | BK_SUM-SUM | 6.230871e-09 |
| | Cu_ZnSOD | BK<br>SUM<br>BK+SUM<br>Untreated | 4.06e-10 | BK-Untreated | 1.193918e-06 |
| | | | | SUM-Untreated | 7.801637e-09 |
| | | | | BK_SUM-Untreated | 4.833769e-10 |
| | | | | SUM-BK | 4.831935e-02 |
| | | | | BK_SUM-BK | 8.523179e-04 |
| | | | | BK_SUM-SUM | 2.943805e-01 |

| CAT | | Anova | | Tukey - posthock test | |
|---|---|---|---|---|---|
| | | groups | *p-value* | compared gropus | *p-value* |
| | Without_antagonists | BK<br>SUM<br>BK+SUM<br>Untreated | 1.04e-08 | BK-Untreated<br>SUM-Untreated<br>BK_SUM-Untreated<br>SUM-BK<br>BK_SUM-BK<br>BK_SUM-SUM | 5.801772e-09<br>1.295126e-02<br>2.975734e-05<br>2.823485e-06<br>1.013560e-03<br>6.039730e-02 |
| | With_HOE_140 | BK<br>SUM<br>BK+SUM<br>Untreated | 4.56e-05 | BK-Untreated<br>SUM-Untreated<br>BK_SUM-Untreated<br>SUM-BK<br>BK_SUM-BK<br>BK_SUM-SUM | 8.298762e-05<br>8.313544e-04<br>3.322120e-01<br>7.378534e-01<br>4.216854e-03<br>3.987633e-02 |
| | With_ Eticlopride | BK<br>SUM<br>BK+SUM<br>Untreated | 2.09e-07 | BK-Untreated<br>SUM-Untreated<br>BK_SUM-Untreated<br>SUM-BK<br>BK_SUM-BK<br>BK_SUM-SUM | 8.026403e-07<br>4.241229e-01<br>1.998247e-05<br>1.917193e-05<br>4.135816e-01<br>6.314450e-04 |

| | | | Anova | Tukey - posthock test | |
|---|---|---|---|---|---|
| | | groups | *p-value* | compared gropus | *p-value* |
| pNOS3_NOS3 | 2min_pNOS3_NOS3 | BK<br>SUM<br>BK+SUM<br>Untreated | 1.5e-08 | BK-Untreated | 7.201554e-09 |
| | | | | SUM-Untreated | 2.737548e-06 |
| | | | | BK_SUM-Untreated | 1.665514e-05 |
| | | | | SUM-BK | 1.798429e-02 |
| | | | | BK_SUM-BK | 2.554091e-03 |
| | | | | BK_SUM-SUM | 8.197531e-01 |
| | 5min_pNOS3_NOS3 | BK<br>SUM<br>BK+SUM<br>Untreated | 9.57e-12 | BK-Untreated | 3.949019e-01 |
| | | | | SUM-Untreated | 2.539412e-06 |
| | | | | BK_SUM-Untreated | 1.914713e-11 |
| | | | | SUM-BK | 7.659734e-05 |
| | | | | BK_SUM-BK | 1.536371e-10 |
| | | | | BK_SUM-SUM | 1.976721e-06 |

| | | Anova | | Tukey - posthock test | |
|---|---|---|---|---|---|
| | | groups | *p-value* | compared gropus | *p-value* |
| NO | 30minNO | BK<br>SUM<br>BK+SUM<br>Untreated | 0.00276 | BK-Untreated<br>SUM-Untreated<br>BK_SUM-Untreated<br>SUM-BK<br>BK_SUM-BK<br>BK_SUM-SUM | 0.086744515<br>0.007476693<br>0.003469724<br>0.660109479<br>0.458185818<br>0.986099658 |
| | 60minNO | BK<br>SUM<br>BK+SUM<br>Untreated | 1.28e-11 | BK-Untreated<br>SUM-Untreated<br>BK_SUM-Untreated<br>SUM-BK<br>BK_SUM-BK<br>BK_SUM-SUM | 7.279702e-02<br>3.052392e-05<br>6.930326e-10<br>1.656410e-07<br>2.070077e-11<br>4.410506e-05 |

| | | Anova | | Tukey - posthock test | |
|---|---|---|---|---|---|
| | | groups | *p-value* | compared gropus | *p-value* |
| Interleukin_6 | Without_TNF_a | | 0.551 | BK-Untreated | 0.7061796 |
| | | BK | | SUM-Untreated | 0.9998631 |
| | | SUM | | BK_SUM-Untreated | 0.8001583 |
| | | BK+SUM | | SUM-BK | 0.6633997 |
| | | Untreated | | BK_SUM-BK | 0.9982521 |
| | | | | BK_SUM-SUM | 0.7614603 |
| | With_TNF_a | | 1.35e-15 | BK-Untreated | 2.980323e-10 |
| | | BK | | SUM-Untreated | 2.566915e-04 |
| | | SUM | | BK_SUM-Untreated | 6.044850e-08 |
| | | BK+SUM | | SUM-BK | 1.496581e-13 |
| | | Untreated | | BK_SUM-BK | 2.242651e-14 |
| | | | | BK_SUM-SUM | 8.195509e-03 |
| | With_TNF_a _HOE_140 | | 0.185 | BK-Untreated | 0.1546846 |
| | | BK | | SUM-Untreated | 0.5344572 |
| | | SUM | | BK_SUM-Untreated | 0.8915669 |
| | | BK+SUM | | SUM-BK | 0.8404375 |
| | | Untreated | | BK_SUM-BK | 0.4653159 |
| | | | | BK_SUM-SUM | 0.9144432 |
| | With_TNF_a _Eticlopride | | 4.01e-14 | BK-Untreated | 4.349854e-13 |
| | | BK | | SUM-Untreated | 9.702817e-01 |
| | | SUM | | BK_SUM-Untreated | 1.930783e-06 |
| | | BK+SUM | | SUM-BK | 2.456924e-13 |
| | | Untreated | | BK_SUM-BK | 8.711774e-08 |
| | | | | BK_SUM-SUM | 6.933470e-07 |

| | | Anova | | Tukey - posthock test | |
|---|---|---|---|---|---|
| | | groups | *p-value* | compared gropus | *p-value* |
| **Apoptosis protein** | 24h_Bcl_2_Bax | BK<br>SUM<br>BK+SUM<br>Untreated | 1.85e-15 | BK-Untreated<br>SUM-Untreated<br>BK_SUM-Untreated<br>SUM-BK<br>BK_SUM-BK<br>BK_SUM-SUM | 0.4602546<br>0.5891874<br>0.0000000<br>0.9961957<br>0.0000000<br>0.0000000 |
| | 24h_Bcl_x_Bax | BK<br>SUM<br>BK+SUM<br>Untreated | 1.28e-10 | BK-Untreated<br>SUM-Untreated<br>BK_SUM-Untreated<br>SUM-BK<br>BK_SUM-BK<br>BK_SUM-SUM | 8.148886e-01<br>1.000000e+00<br>2.366233e-09<br>8.161142e-01<br>6.307957e-10<br>2.357360e-09 |
| | 6h_Bcl_2_Bax | BK<br>SUM<br>BK+SUM<br>Untreated | <2e-16 | BK-Untreated<br>SUM-Untreated<br>BK_SUM-Untreated<br>SUM-BK<br>BK_SUM-BK<br>BK_SUM-SUM | 1.645705e-09<br>4.832921e-10<br>4.152234e-14<br>2.198242e-14<br>1.920686e-14<br>1.828931e-07 |
| | 6h_Bcl_x_Bax | BK<br>SUM<br>BK+SUM<br>Untreated | 1.84e-08 | BK-Untreated<br>SUM-Untreated<br>BK_SUM-Untreated<br>SUM-BK<br>BK_SUM-BK<br>BK_SUM-SUM | 1.013640e-01<br>1.577818e-03<br>2.550105e-06<br>7.233849e-06<br>2.746329e-08<br>3.657388e-02 |

| | | Anova | | Tukey - posthock test | |
|---|---|---|---|---|---|
| | | groups | *p-value* | compared gropus | *p-value* |
| **Caspase 3_7** | Without_TNF_a | BK<br>SUM<br>BK+SUM<br>Untreated | 0.00111 | BK-Untreated | 0.763252238 |
| | | | | SUM-Untreated | 0.545719758 |
| | | | | BK_SUM-Untreated | 0.046222107 |
| | | | | SUM-BK | 0.983512551 |
| | | | | BK_SUM-BK | 0.004033053 |
| | | | | BK_SUM-SUM | 0.001507317 |
| | With_TNF_a | BK<br>SUM<br>BK+SUM<br>Untreated | <2e-16 | BK-Untreated | 1.286524e-09 |
| | | | | SUM-Untreated | 2.013828e-01 |
| | | | | BK_SUM-Untreated | 2.079931e-10 |
| | | | | SUM-BK | 9.558798e-12 |
| | | | | BK_SUM-BK | 1.002531e-13 |
| | | | | BK_SUM-SUM | 3.966948e-08 |

| | | Anova | | Tukey - posthock test | |
|---|---|---|---|---|---|
| | | groups | *p-value* | compared gropus | *p-value* |
| **Endothelin1** | Without_TNF_a | BK<br>SUM<br>BK+SUM Untreated | <2e-17 | BK-Untreated | 1.713095e-06 |
| | | | | SUM-Untreated | 4.808566e-03 |
| | | | | BK_SUM-Untreated | 3.720357e-13 |
| | | | | SUM-BK | 2.419932e-10 |
| | | | | BK_SUM-BK | 0.000000e+00 |
| | | | | BK_SUM-SUM | 6.081829e-10 |
| | With_TNF_a | BK<br>SUM<br>BK+SUM Untreated | <2e-16 | BK-Untreated | 0.0000000 |
| | | | | SUM-Untreated | 0.0000000 |
| | | | | BK_SUM-Untreated | 0.0000000 |
| | | | | SUM-BK | 0.0000000 |
| | | | | BK_SUM-BK | 0.0000000 |
| | | | | BK_SUM-SUM | 0.0000000 |
