# Supplementary material for: Influence of bradykinin B2 receptor and dopamine D2 receptor on the oxidative stress, inflammatory response, and apoptotic process in human endothelial cells
Source: PLoS One. 2018 Nov 14;13(11):e0206443. doi: 10.1371/journal.pone.0206443 (PMC6241119; doi:10.1371/journal.pone.0206443)
Supplement: S2 Table — Normalized original results from at least three experiments are presented in separate tables. (PDF) [file pone.0206443.s002.pdf]

ROS		Without antagonists					With HOE 140					With Eticlopride					With Apocynin			
		Untreated	BK	SUM	BK+SUM		Untreated	BK	SUM	BK+SUM		Untreated	BK	SUM	BK+SUM		Untreated	BK	SUM	BK+SUM
1 exp.	1	96,9084516	222,69328	110,0499	172,4947	1	100,32833	122,1448	100,8888	111,2772	1	103,378	276,6546	100,0351	231,6492	1	97,22726	108,785	103,8799	75,11496
	2	103,091548	270,67283	102,57301	220,0394	2	99,819935	120,9149	108,238	113,6632	2	98,27031	266,2031	119,9872	212,5588	2	101,7842	102,3976	86,01913	88,62673
	3	100	198,95115	106,311455	162,9869	3	99,851737	113,4871	107,4033	109,8161	3	98,35165	241,6308	126,122	238,1707	3	100,9886	102,0148	94,49532	82,19411
2 exp.	1	110,273785	288,39853	117,958025	156,4201	1	99,808433	117,0084	123,7529	109,5825	1	95,28783	212,7786	104,3957	233,2783	1	91,23318	96,8611	102,3658	93,76768
	2	91,106378	344,06355	102,429267	145,483	2	99,300278	139,1303	114,8622	126,4521	2	101,5751	194,9958	117,5176	191,65	2	96,71284	87,39084	92,53225	82,12367
	3	98,6198373	269,367	106,993584	187,5511	3	100,89129	156,9795	102,4723	141,8595	3	103,137	183,1968	103,2816	197,6247	3	112,054	75,56671	103,2085	72,7657
3 exp.	1	105,349583	233,16641	101,211773	188,3304	1	105,34474	128,252	105,9332	116,841	1	108,5469	290,4874	105,0369	243,2316	1	103,0609	114,2242	109,0739	78,87071
	2	99,5626586	306,11579	106,47281	186,0744	2	96,32127	136,3477	112,565	123,9231	2	99,54364	191,0959	115,1673	187,817	2	95,74571	85,64302	90,68161	80,4812
	3	95,0877582	286,8759	107,178863	187,2024	3	98,85322	112,3522	106,3293	108,718	3	96,38461	239,2145	124,8608	235,7889	3	100,8876	100,9947	93,55037	81,37217
4 exp.	1	101,229447	202,63007	101,747765	151,3148															
	2	88,2777795	206,41821	115,617567	168,0685															
	3	110,492774	217,03223	111,082486	152,286															

		MnSOD					Cu/ZnSOD			
		Untreated	BK	SUM	BK+SUM		Untreated	BK	SUM	BK+SUM
1 exp.	1	100	122,6192	114,9364	80,231	1	100	121,2828	130,3597	132,3255
2 exp.	1	100	132,6977	111,6667	89,67555	1	100	132,8234	143,6573	147,0007
3 exp.	1	100	132,2609	121,2752	93,72671	1	100	128,455	138,7516	152,9468

catalase		Without antagonists					Withi HOE 140					With Eticlopride			
		Untreated	BK	SUM	BK+SUM		Untreated	BK	SUM	BK+SUM		Untreated	BK	SUM	BK+SUM
1 exp.	1	100	216,5342	155,4623	122,8133	1	100	159,3002	135,2707	104,8602	1	100	242,3182	121,2048	211,6643
2 exp.	1	100	238,6765	137,3693	182,9758	1	100	119,1066	130,3067	124,7676	1	100	245,4999	140,1864	198,3315
3 exp.	1	100	185,3693	134,3632	145,3536	1	100	130,0051	123,2576	103,575	1	100	158,8078	106,9427	167,446
4 exp.	1	100	214,3574	127,069	189,6531										
5 exp.	1	100	182,142	118,0644	167,4057										

		pNOS3/NOS3 - 2 min					pNOS3/NOS3 - 5 min			
		**Untreated**	**BK**	**SUM**	**BK+SUM**		**Untreated**	**BK**	**SUM**	**BK+SUM**
1 exp.	1	100	177,3797	153,6946	147,6678	1	100	113,1034	167,3749	223,1793
2 exp.	1	100	189,8652	166,4239	157,3902	1	100	108,9749	153,7224	205,3006
3 exp.	1	100	156,1441	133,8475	130,4889	1	100	121,221	175,4673	268,0064

		NO - 30 min					NO - 60 min			
		Untreated	BK	SUM	BK+SUM		Untreated	BK	SUM	BK+SUM
1 exp.	1	100,66135	174,8298	209,2005	236,1603	1	95,7340165	70,07129	184,0833	253,8204
	2	109,356156	168,5275	236,5104	241,3538	2	94,3305859	76,45355	178,102	269,5255
	3	89,9824937	168,936	235,6351	237,269	3	109,935398	89,21809	158,3203	248,9753
2 exp.	1	88,9345045	134,1035	134,8768	143,6414	1	113,208515	78,14824	178,456	202,6417
	2	106,893259	139,4883	143,7273	140,3762	2	88,7407027	65,47833	147,0121	213,3111
	3	104,172236	149,5131	129,2343	135,5643	3	98,0507823	50,62837	152,7315	198,1021
3 exp.	1	100,862673	175,1795	209,6189	236,6326	1	96,1169526	70,21143	184,4515	254,3281
	2	107,169033	165,157	231,7802	236,5267	2	93,38728	74,92448	174,54	264,135
	3	91,7821436	169,2739	236,1064	237,7436	3	110,265204	89,39652	158,637	249,4732

IL-6		Without TNF-a					With TNF-a					With TNF-a i HOE 140					With TNF-a i Eticlopride			
		Untreated	BK	SUM	BK+SUM		Untreated	BK	SUM	BK+SUM		Untreated	BK	SUM	BK+SUM		Untreated	BK	SUM	BK+SUM
1 exp.	1	91,242938	98,0226	102,5424	100,8475	1	94,977169	134,3988	86,60578	76,56012	1	100,045998	106,53174	103,9098	107,2217	1	97,18115	130,8687616	97,45841	114,6488
	2	101,12994	101,1299	106,2147	98,16384	2	104,87062	129,2237	86,60578	80,3653	2	99,9080037	104,46182	100,736	103,0819	2	104,5287	131,4232902	102,4492	119,5009
	3	107,62712	97,17514	105,0847	91,24294	3	100,15221	132,4201	91,62861	83,10502	3	100,045998	102,66789	96,18215	106,2557	3	98,2902	143,0683919	98,15157	123,244
2 exp.	1	105,44707	98,3556	92,49743	94,03905	1	94,869035	119,268	87,40581	74,91927	1	98,6610289	98,520085	109,0909	99,08386	1	99,09154	134,0321454	101,8868	114,7449
	2	91,109969	89,10586	92,18911	88,64337	2	107,92967	126,0136	89,84571	73,9146	2	94,1508104	109,65469	104,8626	96,82875	2	102,3061	138,7840671	90,56604	120,0559
	3	103,44296	95,889	102,8263	109,9178	3	104,91568	130,3193	89,98924	81,37783	3	109,372798	104,58069	99,08386	97,53347	3	96,57582	149,6855346	102,7254	118,0992
						4	92,285612	124,7219	81,09078	80,66021	4	97,8153629	109,3728	108,3862	101,6209	4	102,0266	141,7190776	98,95178	112,369
3exp.	1	101,22919	94,42138	88,79753	90,27749	1	106,25332	133,5802	97,89451	83,90958	1	103,59408	103,44609	114,5455	104,0381	1	102,0402	137,4121996	102,3313	120,3812
	2	97,084746	97,08475	101,9661	94,23729	2	103,86652	129,0161	89,08934	80,56405	2	98,9089236	103,4172	99,72861	102,0511	2	103,4834	130,1090573	101,4247	118,3059
	3	102,24576	92,31638	99,83051	86,68079	3	90,4399	122,2275	79,46896	79,047	3	98,0450782	100,61454	94,25851	104,1306	3	96,3244	140,207024	96,18854	120,7791

		Bcl-2/Bax - 6					Bcl-x/Bax - 6h			
		Untreated	BK	SUM	BK+SUM		Untreated	BK	SUM	BK+SUM
1 exp.	1	100	65,00494	140,0619	163,6328	1	100	71,28833	109,4365	157,0846
2 exp.	1	100	71,51303	134,6039	155,746	1	100	91,3461	139,4218	162,2289
3 exp.	1	100	70,2739	125,2436	150,8946	1	100	70,30335	171,054	181,6625

		Bcl-2/Bax - 24h					Bcl-x/Bax - 24h			
		Untreated	BK	SUM	BK+SUM		Untreated	BK	SUM	BK+SUM
1 exp.	1	100	92,31614	97,9781	215,9259	1	100	79,85486	106,0862	253,4331
2 exp.	1	100	98,14725	96,46114	184,6605	1	100	98,01214	99,72191	186,6035
3 exp.	1	100	87,90939	87,09923	188,7376	1	100	95,99093	94,11958	194,4514

caspase 3/7

		Without TNF-a					With TNF-a			
		Untreated	BK	SUM	BK+SUM		Untreated	BK	SUM	BK+SUM
1 exp.	1	103,044511	109,9526	102,6887	85,61518	1	99,3638496	121,4895	98,08597	84,75752
	2	96,9554894	102,909	115,5228	80,4158	2	96,2170649	125,2984	102,4857	81,93982
	3	100	106,4308	109,1057	83,01549	3	104,419085	118,1944	103,6591	77,80963
2 exp.	1	95,350291	101,1895	105,746	104,5193	1	99,8391174	116,1276	91,52344	73,47394
	2	104,649709	109,5757	106,4737	99,12982	2	95,5965366	118,4628	88,42488	75,32215
	3	100	105,3826	106,1099	101,8246	3	104,564346	120,532	87,13298	76,73605
3 exp.	1	93,250417	103,73	89,77593	84,35466	1	103,514981	123,1037	98,41425	83,56718
	2	105,299778	91,9664	100,652	87,31975	2	90,3060503	119,1846	95,95101	74,39132
	3	101,449805	96,76395	102,1521	95,45997	3	106,178969	116,8748	94,48714	78,45136

Endothelin-1		Without TNF-a					With TNF-a			
		Untreated	BK	SUM	BK+SUM		Untreated	BK	SUM	BK+SUM
1 exp.	1	95,8168903	108,2873	91,87056	76,5588	1	97,4658869	168,8109	52,24172	71,1501
	2	104,18311	112,5493	95,65904	77,50592	2	102,534113	170,1754	50,09747	73,29435
2 exp.	1	94,0805051	113,4964	92,65983	80,66298	1	103,508772	168,4211	52,24172	71,73489
	2	105,919495	110,6551	94,86977	73,24388	2	96,4912281	166,6667	47,75828	71,92982
3 exp.	1	96,0085241	108,5039	92,0543	76,71192	1	97,6608187	169,1485	52,3462	71,2924
	2	104,8603	109,5485	93,92107	72,51144	2	103,245614	178,3333	51,10136	76,96491
